# A Novel JAK1 Mutant Breast Implant-Associated Anaplastic Large Cell Lymphoma Patient-Derived Xenograft Fostering Pre-Clinical Discoveries

**DOI:** 10.3390/cancers12061603

**Published:** 2020-06-17

**Authors:** Danilo Fiore, Luca Vincenzo Cappelli, Paul Zumbo, Jude M. Phillips, Zhaoqi Liu, Shuhua Cheng, Liron Yoffe, Paola Ghione, Federica Di Maggio, Ahmet Dogan, Inna Khodos, Elisa de Stanchina, Joseph Casano, Clarisse Kayembe, Wayne Tam, Doron Betel, Robin Foa’, Leandro Cerchietti, Raul Rabadan, Steven Horwitz, David M. Weinstock, Giorgio Inghirami

**Affiliations:** 1 Department of Pathology and Laboratory Medicine, Weill Cornell Medicine, New York, NY 10065, USA; danilofiore87@gmail.com (D.F.); lvc4001@med.cornell.edu (L.V.C.); shc2019@med.cornell.edu (S.C.); liy2010@med.cornell.edu (L.Y.); fede_dimaggio@yahoo.it (F.D.M.); joc9235@nyp.org (J.C.); clk2010@med.cornell.edu (C.K.); wtam@med.cornell.edu (W.T.); 2 Department of Translational and Precision Medicine, Sapienza University of Rome, 00161 Rome, Italy; rfoa@bce.uniroma1.it; 3 Applied Bioinformatics Core, Weill Cornell Medicine, New York, NY 10065, USA; pzumbo@zoho.com; 4 Department of Medicine, Hematology-Oncology, Weill Cornell Medicine and the New York Presbyterian Hospital, New York, NY 10065, USA; jup2021@med.cornell.edu (J.M.P.); lec2010@med.cornell.edu (L.C.); 5 Department of Systems Biology and Biomedical Informatics, Columbia University, New York, NY 10032, USA; zl2495@cumc.columbia.edu (Z.L.); rr2579@cumc.columbia.edu (R.R.); 6 Department of Medicine, Memorial Sloan-Kettering Cancer Center, 1275 York Avenue, New York, NY 10065, USA; ghionep@mskcc.org (P.G.); horwitzs@mskcc.org (S.H.); 7 Department of Molecular Medicine and Medical Biotechnologies, University of Naples Federico II, 80131 Naples, Italy; 8 Departments of Pathology and Laboratory Medicine, Memorial Sloan-Kettering Cancer Center, 1275 York Avenue, New York, NY 10065, USA; dogana@mskcc.org; 9 Molecular Pharmacology Program, Memorial Sloan Kettering Cancer Center, New York, NY 10065, USA; khodosi@mskcc.org (I.K.); destance@mskcc.org (E.d.S.); 10 Department of Medicine and Institute for Computational Biomedicine, Weill Cornell Medicine, New York, NY 10065, USA; dob2014@med.cornell.edu; 11 Department of Medical Oncology, Dana-Farber Cancer Institute and Harvard Medical School, Boston, MA 02215, USA; DavidM_Weinstock@dfci.harvard.edu

**Keywords:** patient-derived tumor xenograft, JAK/STAT pathway, pre-clinical model, drug discovery, precision medicine

## Abstract

Breast implant-associated lymphoma (BIA-ALCL) has recently been recognized as an independent peripheral T-cell lymphoma (PTCL) entity. In this study, we generated the first BIA-ALCL patient-derived tumor xenograft (PDTX) model (IL89) and a matching continuous cell line (IL89_CL#3488) to discover potential vulnerabilities and druggable targets. We characterized IL89 and IL89_CL#3488, both phenotypically and genotypically, and demonstrated that they closely resemble the matching human primary lymphoma. The tumor content underwent significant enrichment along passages, as confirmed by the increased variant allele frequency (VAF) of mutations. Known aberrations (JAK1 and KMT2C) were identified, together with novel hits, including PDGFB, PDGFRA, and SETBP1. A deep sequencing approach allowed the detection of mutations below the Whole Exome Sequencing (WES) sensitivity threshold, including JAK1G1097D, in the primary sample. RNA sequencing confirmed the expression of a signature of differentially expressed genes in BIA-ALCL. Next, we tested IL89’s sensitivity to the JAK inhibitor ruxolitinib and observed a potent anti-tumor effect, both in vitro and in vivo. We also implemented a high-throughput drug screening approach to identify compounds associated with increased responses in the presence of ruxolitinib. In conclusion, these new IL89 BIA-ALCL models closely recapitulate the primary correspondent lymphoma and represent an informative platform for dissecting the molecular features of BIA-ALCL and performing pre-clinical drug discovery studies, fostering the development of new precision medicine approaches.

## 1. Introduction

Breast implant-associated anaplastic large cell lymphoma (BIA-ALCL) is a rare disease that has recently been categorized by the World Health Organization (WHO) as an independent subtype of peripheral T-cell lymphoma (PTCL) [1]. As of July 2019, a total of 573 cases have been identified within the United States [2,3], with 33 deaths reported. The calculated risk of BIA-ALCL incidence has been related to the patient’s age (1 in 35,000 at 50 years and 1 in 7000 at 75 years) [4] and recent data indicate that the lifetime risk of BIA-ALCL among women with breast implants is estimated to be 1 in 355 women exposed [5]. The majority of patients manifest either seroma and/or in situ disease, both of which are associated with an excellent prognosis (89% overall survival rate at 5 years from the diagnosis) [6]. However, infiltrative disease, associated with the worst prognosis, has been described [7]. Despite its rarity, the excellent prognosis, and incomplete reporting, there is an increasing interest in investigating BIA-ALCL incidence, risk factors, and outcome, mainly dictated by the evidence that breast augmentation is the number-one cosmetic procedure performed in the United States [8]. Recently, a crucial role of cytokine signaling, the JAK/STAT pathway, and epigenetic modifier deregulation were reported to contribute to the pathogenesis of BIA-ALCL [7,9,10,11,12,13,14,15,16]. However, scientific progress has been somewhat hampered by the lack of informative pre-clinical models.

Patient-derived tumor xenografts (PDTX) represent one of the most promising platforms for modeling human cancer and its complexity. PDTX can closely recapitulate several features of the matching donor tumors and remain relatively stable along passages, rendering them an ideal tool to serve as robust and predictive models in oncology [17,18,19,20]. The relationship between human tumors and mouse host elements of PDTX is known to play a relevant role in successful engraftment, growth, and responses to therapy [21]. Therefore, PDTX provide a powerful tool for conducting more reliable pre-clinical investigations [20,22].

Here, we describe the generation and characterization of a newly established BIA-ALCL PDTX model. We demonstrate that our model closely mimics the corresponding patient’s primary sample, sharing all of the main putative driver mutations (including the JAK1 G1097D alteration), as well as most of its transcriptomic signature. The PDTX model was then used to test pre-clinical approaches, such as the sensitivity to JAK inhibitor and high-throughput drug screening (HTS) discovery platforms. Overall, we demonstrated the potential of pre-clinical targeted therapeutic interventions in a new PDTX model of BIA-ALCL, fostering patient-tailored approaches and precision medicine.

## 2. Results

### 2.1. Clinical Data and BIA-ALCL PDTX Model Establishment

The donor patient carried a textured implant and had a prior history of breast cancer. The mass presented with a tumor-type BIA-ALCL, stage T1AN0M0 (MD Anderson classification), with a seroma and some lymphoid aggregates on the lumen of the capsule, without blood involvement. She underwent surgical removal of the implant with complete capsulectomy, achieving a complete clinical remission. The main clinical and pathological features of the patient are summarized in Table 1.

**Figure 1:**
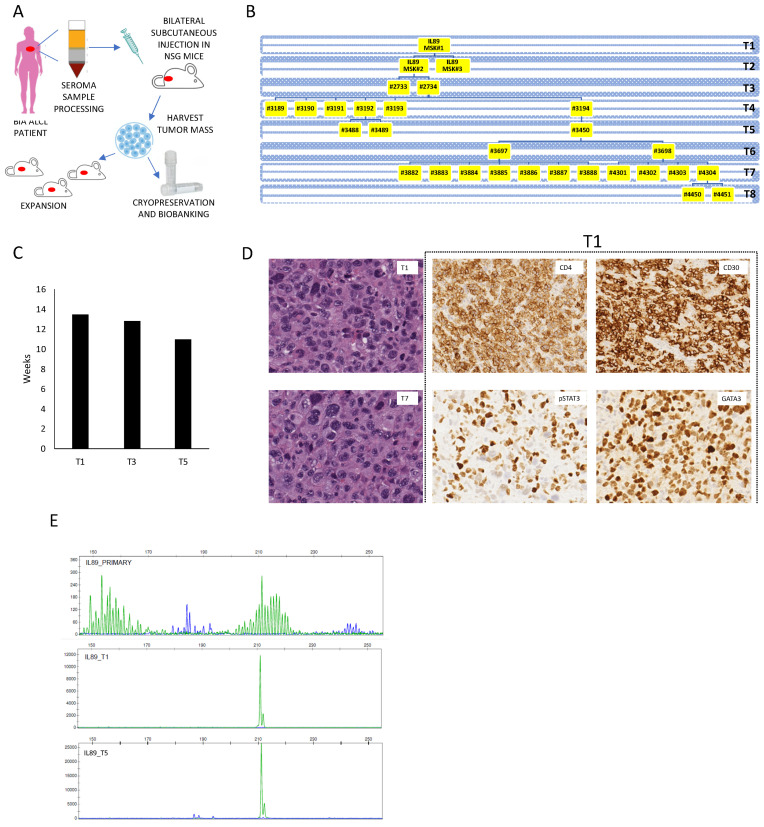
IL89 patient-derived tumor xenograft (PDTX) generation and phenotypical characterization. (**A**) Schematic PDTX generation and expansion workflow. The patient’s material was processed and implanted in immunocompromised mice. Once engrafted, tumor-grafted samples were expanded and extensively bio-banked. (**B**) PDTX engraftment and serial tumor propagation in NOD.Cg-Prkdcscid Il2rgtm1Wjl/SzJ (NSG) mice. Intersecting lines define the relationship between different tumors. (**C**) Relative IL89 PDTX time of engraftment expressed in weeks required to detect a palpable mass over passages T1-T3-T5 (fitness). (**D**) Histology micrografts on IL89 PDTX show strong CD30 membranous staining. Left panels correspond to representative H&E of T1 and T7 PDTX samples (40×). Immunohistochemical stains with the indicated antibodies were carried out (anti-CD4 and -CD30 [20×] and anti-pSTAT3 and -GATA3 [40×]). (**E**) Analysis of the T-cell receptor (TCR) gamma specific rearrangement clonality in the IL89 diagnostic sample and in the corresponding PDTX after one and five passages (T1 and T5).

Currently, there is a lack of pre-clinical models for studying BIA-ALCL. To the best of our knowledge, only three continuous BIA-ALCL cell lines have been developed (namely TLBR1-2-3) [20,23]. These models have real advantages and have allowed important advances in the understanding of BIA-ALCL [12,16,24,25]; however, they display limitations, such as impaired cell–cell and cell–matrix interactions. Moreover, putative clonal selections may occur, along with the establishment of cell lines, determining a potentially incomplete representation of the natural lymphoma heterogeneity [26]. Compared to other PTCL entities, BIA-ALCL grow within a rich host background, which acts as a scaffold to mount a dynamic pro-inflammatory microenvironment defined by a panel of active cytokines crucial for sustaining tumor features [27,28,29]. To overcome these limitations, we established a BIA-ALCL PDTX model (from now on referred to as IL89). A seroma sample was obtained from the treatment-naïve patient and promptly processed to obtain a mononuclear cell suspension. Neoplastic cells were mixed 1:1 in Matrigel and implanted into NOD Cg-Prkdcscid B2mtm1Unc Il2rgtm1Wjl/SzJ mice (β2 ko NSG), proven to be a favorable recipient with a negligible rate of graft-versus-host disease (GVHD) [30]. One mouse was implanted subcutaneously with the primary diagnostic sample and engrafted. The generated PDTX, which emerged ≈13 weeks post-implantation, were passaged several times in mice (PDTX line expansion) and extensively cryopreserved (PDTX line biobanking) (Figure 1A).

The IL89 PDTX line was successfully propagated in mice along eight different passages, and various branches of the tree were expanded for a total number of 29 mice (Figure 1B). The initial engraftment time was 13.5 weeks and did not vary considerably among different passages, with a growing pattern that remained mostly stable (Figure 1C).

Mice implanted subcutaneously with IL89 seeds displayed large tumor masses with back-to-back cells, presenting high pleomorphism and tumor cells infiltrating the surrounding subcutaneous tissues and muscles. Tumor cells were detected at the site of implantation, and no distant metastases were identified (Figure 1D). Murine stromal elements appeared to be more prominent in the early passages and less evident in T5. Immunophenotypically, the lymphoma cells were positive for CD30 (cytoplasmic with a Golgi pattern) and CD25 and did not express CD3 and CD8, but were positive for CD4, and a subset of the lymphoma cells were granzyme-positive (Figure S1A,B). Strong nuclear signals for GATA3 and pSTAT3 were documented in most of the cells (Figure 1D). This PDTX immunohistochemical phenotype closely matched the reported diagnostic data (Table 1). Overall, these features are in line with those previously reported in other BIA-ALCL patients [31].

These findings were confirmed by flow cytometry (Table S1 and Figure S1B).

Next, to prove the clonal correspondence between the primary sample and the PDTX, we performed T-cell receptor (TCR) gene rearrangement analyses of the diagnostic samples and three different PDTX (T1-T3-T5). The data confirmed the presence of a dominant clone, which became increasingly exclusive within the PDTX samples (Figure 1E), and the data were further confirmed with multiple primers (Figure S1C). These findings demonstrated that the IL89 primary sample included a relatively large number of normal T-cells, as previously described [32], which were lost along with PDTX engraftment.

In summary, we generated a novel BIA-ALCL PDTX recapitulating the histologic and immunophenotypical features of the matched primary lymphoma.

### 2.2. Genomic and Transcriptomic Landscapes of IL89 BIA-ALCL Closely Mimic the Primary Tumor

**Figure 2:**
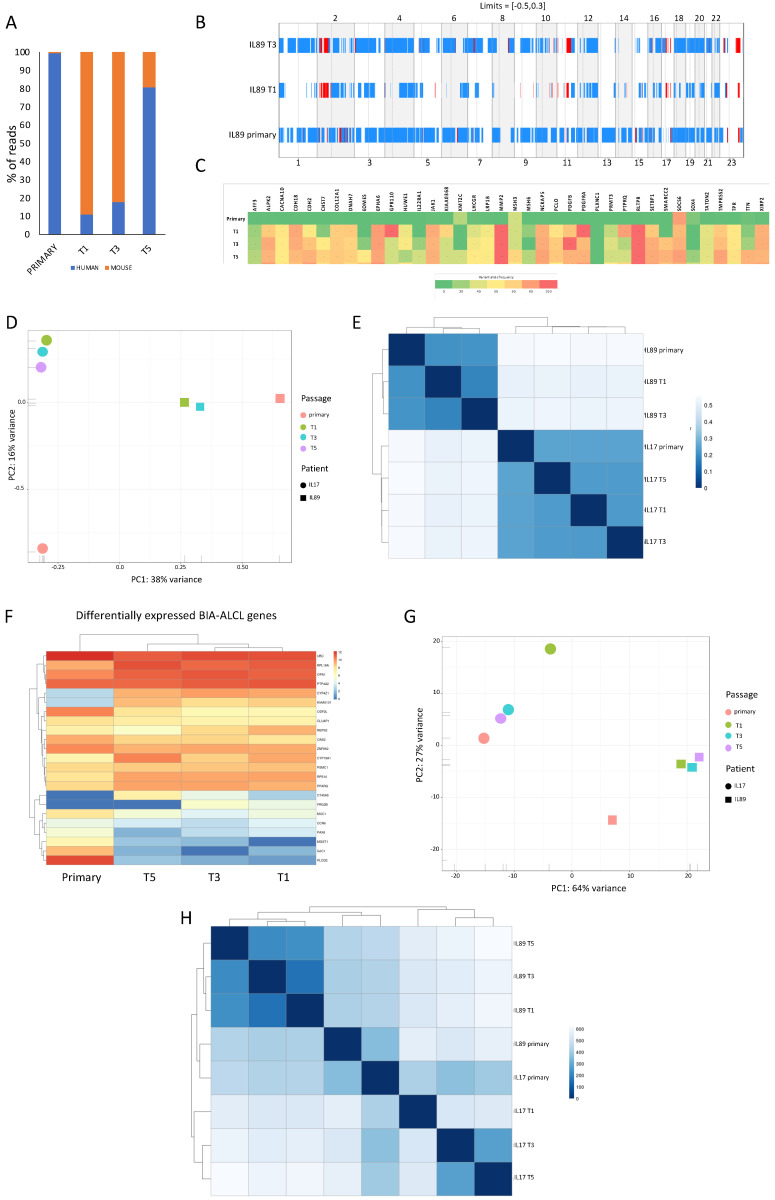
IL89 PDTX molecular landscape. (**A**) The percentage of PDTX human vs. mouse content was calculated by normalizing the number of reads from whole exome sequencing (WES) aligned with the human (hg19) or murine (mm10) genomes. Ambiguous reads were excluded from the analysis. (**B**) Copy number variations (CNV) analysis. Frequency of CNV (Y axis) according to chromosomal regions (X axis) in the IL89 primary sample and PDTX (T1–T3). Blue color indicates the loss of the chromosomal region, while red represents gains. (**C**) Heatmap of the most significantly mutated genes in IL89 detected by WES. The color-code provided is indicative of the variant allele frequency (VAF). (**D**) Principal component analysis based on WES data of IL89 vs. IL17 models shows that the primary genomic fingerprints were maintained in PDTX and that different entities have distinct genomic signatures. (**E**) Unsupervised hierarchical clustering of WES mutational profiles of IL89 vs. IL17. Samples belonging to the same model cluster together. (**F**) RNA expression levels of signatures of BIA-ALCL-associated genes were mostly consistent between the primary sample and the passages. (**G**) Principal component analysis based on total RNA sequencing data of IL89 vs. IL17 models shows that the expression landscapes of the primary tumors were maintained in the respective PDTX models and that the two different entities have distinct transcriptomes. (**H**) Unsupervised hierarchical clustering of total RNA sequencing profiles of IL89 vs. IL17. Samples belonging to the same model cluster.

To further confirm the correspondence of the IL89 PDTX model and its corresponding primary tumor and their molecular landscape, we performed whole exome sequencing (WES) and total RNA sequencing of the patient’s primary tumor sample and the T1, T3, and T5 passages of the PDTX. A matched saliva sample from the patient was included as a standard reference to filter out polymorphisms and non-pathogenic variants from the WES analysis. We firstly focused on tumor purity using a bio-informatic approach to distinguish between human and mouse reads (see Methods) (Figure 2A).

Interestingly, the human content was low in the T1 (~10%), which is a phenotype that slowly changed along the passages. Indeed, at the T5 passage, mouse/human inversion was observed, with human reads reaching ~80% of the total tumor content.

We then dissected the genomic landscape by WES, analyzing both the copy number variation (CNV) and mutational burden. As expected, CNVs were mainly maintained from the primary sample to the PDTX along different passages (Figure 2B). Genome-wide DNA profiling demonstrated a broad range of gains and losses homogenously distributed among all chromosomes. This scenario revealed a marked genomic instability, which is a phenotype shared with many Anaplastic Lymphoma Kinase (ALK)-negative ALCL [33]. To define the global mutational burden, we then focused on the primary tumor sample and the PDTX samples up to passage T3. A broad range of somatic mutations were identified for a total of 734 non-synonymous hits, without any preferential chromosomal distribution (Table S2). Mutations were largely represented by single nucleotide substitutions leading to amino acid changes, namely missense mutations (624/734; 85%), but also included frameshift insertions or deletions (15/734; 2%), non-frameshift insertions or deletions (13/734; 1.8%), nonsense mutations (62/734; 8.4%), and alterations in canonical splice sites (19/734; 2.6%) (Table S3). Using Sorting Intolerant form Tolerant (SIFT) and PolyPhen predictors, we identified a total of 281/734 (38%) that were defined as likely pathogenic by both models. Importantly, the mutation landscape was largely shared between the primary sample and the PDTX model across all passages (Figure 2C). As expected, we found hits in genes described to be mutated in BIA-ALCL [9] (i.e., JAK1, KMT2C, and EOMES) and the variant allele frequency (VAF) of almost all the driver mutations increased from the primary sample to the PDTX (Figure 2C). Of note, we also detected novel alterations (e.g., ALPK2, CDH2, COL12A1, EPH6, GPR110, IL22RA1, KIAA0368 LHCGR, LRP1B, MMP2, PDGFB, PDGFRA, PRMT3, PTPRQ, RLTPR, SETBP1, and SOX4), with a putative pathogenetic role in BIA-ALCL in the PDTX samples. These defects were not detected in the primary sample (Table S4), likely due to the low sensitivity of the WES analysis.

We then compared the mutational profile of our IL89 BIA-ALCL model with that of an ALK-ALCL PDTX model (IL17) developed in our laboratory. As expected, all samples belonging to the IL89 model clustered together by principal component analysis (PCA) and hierarchical clustering based on the Euclidean distances calculated between each sample (Figure 2D,E). Meanwhile, IL17 displayed a genomic fingerprint shared by the primary and corresponding PDTX and distinct from that of IL89. We chose a representative ALK-ALCL model for a comparison to test whether they share similarities and/or whether they gain common features with serial propagation as a result of an increased fitness in the mouse environment. The data indicate that the genomic fingerprints of these models remain mostly constant, and no significant shift was observed as a result of the mouse environmental pressure.

Next, we evaluated the RNA sequencing data and looked for the expression of different genes and their uniformity between the primary sample and the PDTX model (Figure 2F). We selected representative genes by merging the RNA sequencing expression data available in the literature [34] and identified a group of genes that were differentially expressed between BIA-ALCL and at least three different T-cell neoplasm entities (e.g., ALCL, AITL, ALK-ALCL, ALK+ ALCL, and PTCL-NOS). This strategy confirmed the high degree of concordance between the primary sample and the PDTX model. Moreover, we found that most of these genes were substantially over-expressed (Figure 2F), with few down-regulated genes in the PDTX compared to the primary tumor, likely linked to a possible rewiring of tumor cells within the murine environment. Indeed, as indicated above (Figure 2A), along the passages, the ratio of lymphoma/mouse increased, suggesting a reasonable augmented fitness and/or a loss of mouse dependency. We finally compared the expression data of the IL89 model with that of the IL17 ALK-ALCL model, and saw that these two models were distinct in the PCA and the unsupervised correlation matrix (Figure 2G,H).

### 2.3. Clonal Evolution of the IL89 BIA-ALCL PDTX Model

**Figure 3:**
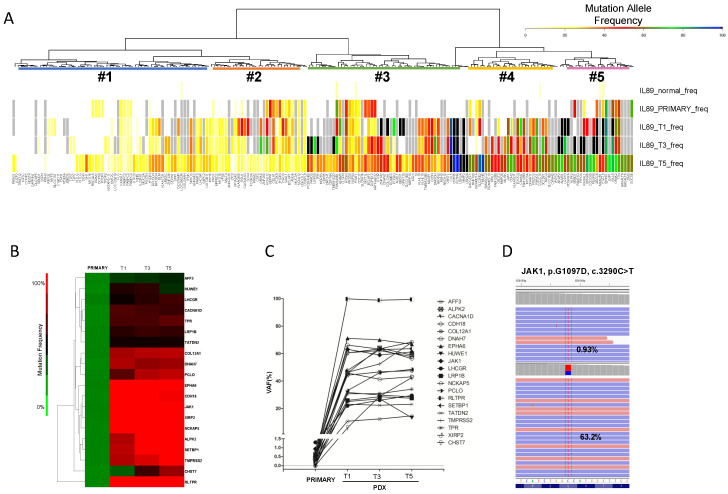
IL89 PDTX clonal evolution in mice. (**A**) Unsupervised hierarchical clustering and relative heatmap of the mutational burden (WES data) of the IL89 diagnostic sample and corresponding PDTX (Passages T1-T3-T5) revealed the presence of five different clusters. The color-code provided is indicative of the VAF. (**B**) Unsupervised hierarchical clustering and relative heatmap of the mutational burden (targeted deep sequencing data) of the IL89 diagnostic sample and corresponding PDTX (passage T1-T3-T5) revealed the presence of five different clusters. The color-code provided is indicative of the VAF. (**C**) Specific VAF enrichment in PDTX (relative to the matched primary sample) of selected mutations analyzed by targeted deep sequencing. (**D**) Manual backtracking of mutations in the primary tumor using deep sequencing data allowed for the identification of the G1097D JAK1 mutation at a very low VAF (0.93%) compared to the PDTX-T1 (63.2%).

Cancers naturally undergo clonal expansion and selection that can also depend on the ecosystem [35,36]. To discover if any change had occurred along with the IL89 PDTX propagation, we decided to first interrogate the mutational landscape of PDTX along multiple passages (T1-T3-T5). Using WES data, we assessed synonymous and non-synonymous mutations with an allelic frequency >5%, and identified five main clusters by unsupervised analysis (Figure 3A).

Interestingly, most of the mutations underwent a VAF increase from the primary sample to the PDTX model, which was also evident along serial passages (T5 displayed the highest VAF). This phenomenon is in line with the findings obtained on tumor purity (Figure 2A), confirming the enrichment of tumor cells with propagation while maintaining the same molecular fingerprint observed in the primary sample.

To further confirm these findings, we implemented a targeted deep sequencing approach to validate the mutations found by WES and observed their evolution along with the primary sample and PDTX passages T1-T3-T5 (Figure 3B). This backtracking approach took advantage of the deeper coverage (mean coverage: >1100) compared to WES for a panel of 538 genes more frequently mutated in PTCL (Table S5 and Figure 3B–D). This strategy confirmed all the WES mutations covered by the targeted library. These included the canonical JAK1 p.G1097D in the primary tumor at a 0.93% VAF, which was then enriched in the PDTX, reaching a 63.2% VAF in passage T1 (Figure 3D, Figure S2A).

Moreover, mutations that were undetectable by WES for the primary sample were successfully identified, suggesting that they already occurred in the original patient’s tumor, but could not be detected because of the lower VAF and limited WES sensitivity. These include LRP1B, COL12A1, SETBP1, EPHA6, RLTPR, ALPK2, and LHCGR (Figure S2B), which may have a pathogenetic role and should be further functionally validated.

### 2.4. JAK/STAT Signaling Represents a Targetable Vulnerability in JAK1-mutant IL89 PDTX

**Figure 4:**
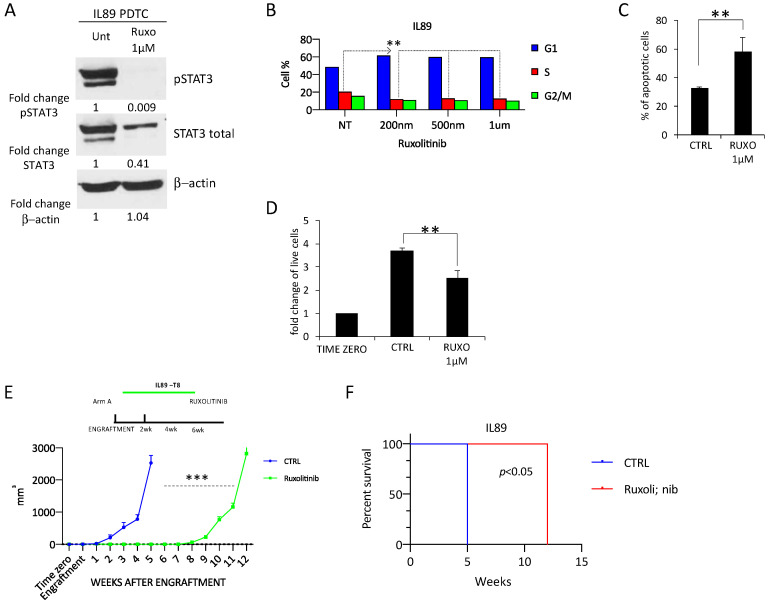
IL89 response to ruxolitinib treatment in vitro and in vivo. (**A**) Ruxolitinib treatment (72 h and1 µM) in IL89 patient-derived tumor cells (PDTC) resulted in a decrease of STAT3 phosphorylation and cell cycle arrest (**B**), an increase of the apoptotic rate (**C**), and a reduction of the cell number (**D**) in vitro. In vivo treatment with ruxolitinib (chow ad libitum) of IL89 PDTX mice determined a strong decrease of tumor growth (**E**) and an increased overall survival (**F**). Data are representative of at least three independent experiments and values are expressed as the average ± standard deviation. *p*-values were calculated using the student *t*-test. ** *p* < 0.01, *** *p* < 0.001, A log-rank test was used to calculate the *p*-value in (**F**).

As PDTX provide a useful tool for studying the functional impact of selected mutations, we validated the therapeutic efficacy of the JAK1/2 inhibitor ruxolitinib, which is a compound currently in phase II clinical trials for the treatment of refractory/relapsed T- or NK-cell lymphoma (NCT029746471 [37]). The overall objective was to define the tumorigenic role of the JAK deregulation in IL89 and gain possible data on the therapeutic efficacy of ruxolitinib in JAK/STAT3 mutated BIA-ALCL. We first demonstrated that PDTX-derived cells (PDTC) cultured in vitro displayed high levels of phosphorylated STAT3 (pSTAT3) (Figure 4A left). Next, we proved that the ruxolitinib treatment markedly reduced STAT3 activation (Figure 4A right, Figure S3A), suggesting that the STAT3 pathway is constitutively active in this model, even without lymphokine supplementation.

The loss of pSTAT3 signaling in IL89 PDTC treated with different doses of ruxolitinib was associated with significant cell cycle arrest, with an accumulation in the G1 phase in vitro (Figure 4B). In parallel, ruxolitinib treatment led to an increase in the number of cells undergoing apoptosis detected by the Annexin V-7AAD assay (Figure 4C), as well as a reduced cell viability, as assessed by a trypan blue cell exclusion assay (Figure 4D).

Although in vitro screenings have led to the discovery of new compounds, many drug platforms do not routinely test the contribution of the host environment. To address this weakness, we expanded our studies in vivo on PDTX IL89. Mice were xenografted and then randomized to either a vehicle or ruxolitinib (Figure 4E). The treatment with ruxolitinib resulted in a potent and significant impairment of tumor growth, with complete remission of the disease in all five treated mice, in the absence of a detectable toxicity (Figure S3B). After 5 weeks, treatment was suspended, and the outcome of mice observed. Approximately 3 weeks after stopping ruxolitinib, all mice relapsed and succumbed to the disease within 12 weeks (Figure 4E). In summary, ruxolitinib treatment significantly reduced the tumor growth and improved the overall survival (OS) in IL89 (Figure 4E,F), even if it did not completely eradicate the disease.

These data demonstrate that the IL89 model can be efficiently used to find and test new potential vulnerabilities in BIA-ALCL.

### 2.5. The IL89-Derived Continuous Cell Line Recapitulates the Corresponding PDTX

As drug discovery programs using PDTX in vivo have been proven to be successful, they require highly specialized units and considerable financial support [38]. Moreover, BIA-ALCL are rare entities, and the generation of representative libraries with a sufficient number of models is an unreachable objective. To address this issue, we generated a novel BIA-ALCL cell line from the IL89 PDTX.

**Figure 5:**
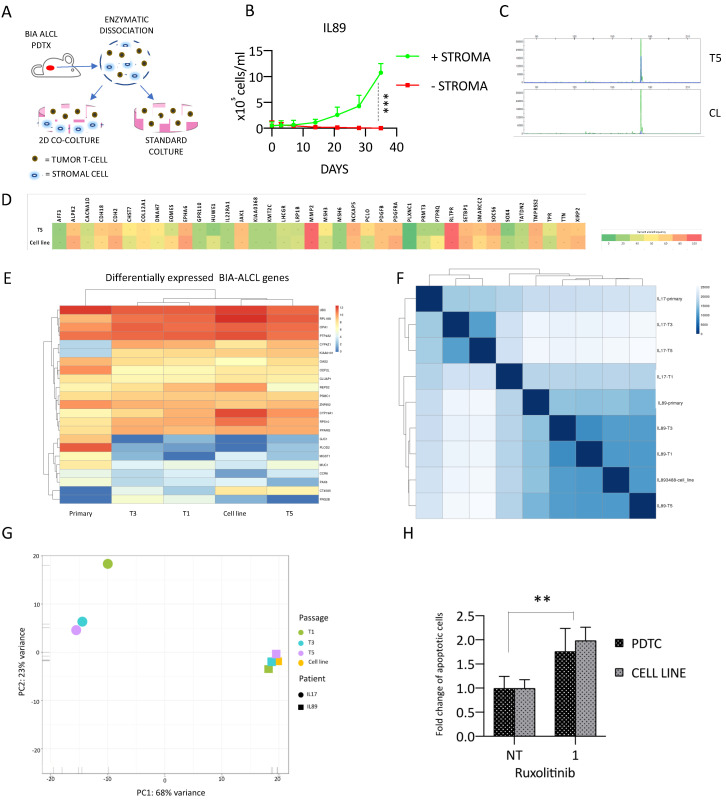
IL89_CL#3488 continuous cell line generation and characterization. (**A**) Schematic IL89_CL#3488 continuous cell line derivation strategy. The patient’s material was digested and cultured overnight. Floating lymphoma cells were isolated and cultured alone or with stromal elements, attached to the plate. (**B**) Growth curve of IL89 cells cultured alone or in the presence of stromal elements. (**C**) TCR gamma specific rearrangement clonality of IL89 PDTX T5 and the IL89_CL#3488 continuous cell line showed the identity of the tumor clone. (**D**) Heatmap of the most significantly mutated genes in IL89 detected by WES shows a similarity between IL89 T5 and the IL89_CL#3488 continuous cell line. The color-code provided is indicative of the VAF. (**E**) RNA expression levels of a breast implant-associated anaplastic large cell lymphoma (BIA-ALCL) gene signature shows a strict association between the IL89 diagnostic sample, PDTX (T1-T3-T5), and the IL89_CL#3488 continuous cell line. (**F**) Unsupervised hierarchical clustering of total RNA sequencing profiles of IL89 (PDTX and cell line) vs, IL17 ALK-ALCL model. Samples of the same models cluster together. (**G**) Principal component analysis based on total RNA sequencing data of IL89 (Primary-PDTX-cell line) vs. IL17 models showed that the expression landscapes of the primary tumors were maintained in the respective PDTX models and that the two different entities have distinct transcriptomes. The IL89_CL#3488 cell line fits in the same cluster as its primary and PDTX-derived passages. (**H**) IL89_CL#3488 recapitulates the ruxolitinib sensitivity of the corresponding PDTC (72 h, 1 µM). Data are representative of at least three independent experiments, and values are expressed as the average ± standard deviation. *p*-values were calculated using the student *t*-test. ** *p* < 0.01, *** *p* < 0.001.

Initially, we reasoned that culturing lymphoma cells with the host murine intra-tumoral elements would sustain/support the survival and/or growth of PDTX cells from IL89-T5. To test this hypothesis, we isolated, by mild digestion of the extracellular matrix, both human and murine cells. Human lymphoma cells were then cultured alone or co-cultured with murine stromal elements (2D co-culture) (Figure 5A).

As depicted in Figure 5B, in these early attempts, the lymphoma cells, lacking host support, died, suggesting that the murine stromal elements could promote their survival and proliferation. This dependency was eventually overcome after ~1.5 months of continuous co-culture. Ultimately, a continuous cell line (IL89_CL#3488) could be effectively expanded in base media (RPMI supplemented with 20% FBS) without lymphokine supplementation and in the absence of murine elements. At present, IL89_CL#3488 has been in culture for more than 2 years.

Once IL89_CL#3488 became stable, we extensively characterized it by comparing its phenotypic and molecular features to the matched IL89 PDTX (T5), demonstrating an identical TCR gene rearrangement (Figure 5C, Figure S4A) and a similar immunophenotypic profile (Figure S4B, Table S6).

Moreover, the genome-wide DNA profiling proved a high concordance without any significant change of the mutational burden. Interestingly, the VAF of the identified genetic defects was also consistent between PDTX and the corresponding IL89_CL#3488 cell line (Figure 5D).

We extended the total RNA sequencing data to the cell line, demonstrating that the IL89_CL#3488 had a similar profile to those corresponding to the PDTX model or the primary sample, based on the BIA-ALCL-associated gene signature previously described (Figure 5E). We then introduced the ALK-ALCL IL17 model as a comparison. Based on total RNA sequencing data, we observed that the IL89_CL#3488 tightly clustered with the corresponding donor PDTX (IL89-T5) and, to a significant extent, with all of the other IL89 PDTX samples, as well as with the primary sample, by both the unsupervised correlation matrix and PCA (Figure 5F,G).

Finally, we demonstrated that IL89_CL#3488 efficiently mimicked the IL89 sensitivity to ruxolitinib, as assessed by the Annexin V-7AAD assay (Figure 5H). After ~2 years, data on the IL89_CL#3488 sensitivity to ruxolitinib have been reproduced and showed a significant loss of pSTAT3 signaling (also confirmed using the pan-JAK1-2-3 inhibitor tofacitinib, Figure S4C,D) and cell cycle arrest leading to an accumulation in the G1 phase (Figure S4E). In parallel, ruxolitinib treatment led to an increase in the number of cells undergoing apoptosis detected by the Annexin V-7AAD assay (Figure S4F), as well as a reduced cell viability, as assessed by a trypan blue cell exclusion assay (Figure S4G).

### 2.6. The IL89-Derived Continuous Cell Line Allows Pre-Clinical Screening

Taking advantage of the newly established cell line, we used IL89_CL#3488 for a new combinatorial pre-clinical approach, with the aim of finding new effective drug combination regimens.

**Figure 6:**
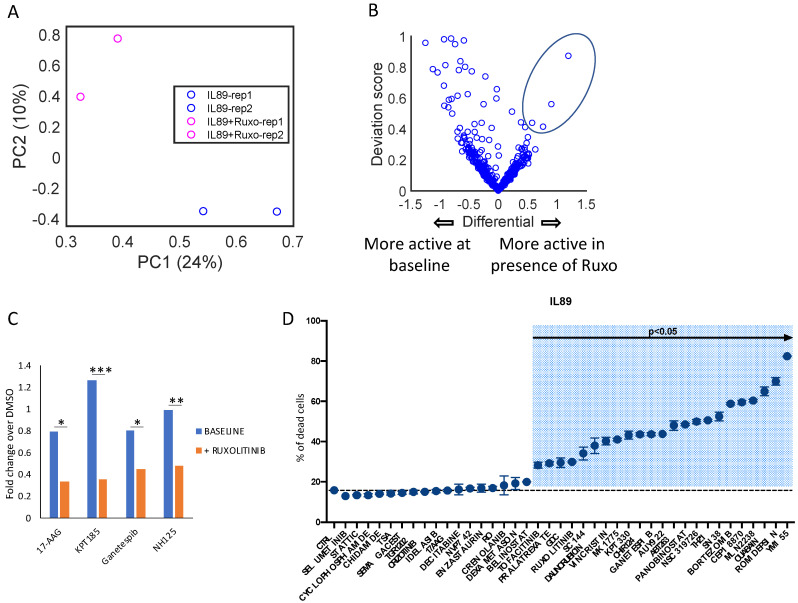
High-throughput screening of IL89_CL#3488 in the presence of ruxolitinib reveals potential synergic combinations. (**A**) Principal component analysis based on the drug response data of IL89_CL#3488 in the absence or presence of ruxolitinib (high-throughput drug screening (HTS) compounds library: 433 drugs, 1 µM, 72 h; ruxolitinib 0.5 µM, 72 h) showed a high degree of concordance among replicates. (**B**) Volcano plots highlighting the overall responses to the drug screening library of IL89_CL#3488 in the absence or presence of ruxolitinib (HTS compounds library: 433 drugs, 1 µM, 72 h; ruxolitinib 0.5 µM, 72 h). Each dot represents a single drug. Dots on the left represent drugs with a higher therapeutic effect in the absence, and on the right in the presence, of ruxolitinib. (**C**) Histogram showing the response of IL89_CL#3488 to 17-AAG, KPT185, ganetespib, and NH125 (1 µM, 72 h) in the absence or presence of ruxolitinib (0.5 µM, 72 h). (**D**) Percentage of IL89_CL#3488 cell death after treatment with a battery of 40 compounds. Standard deviations are reported. Data are representative of at least three independent experiments, and values are expressed as the average ± standard deviation. *p*-values were calculated using the student t-test and corrected for multiple comparisons using the Bonferroni method. * *p* < 0.05, ** *p* < 0.01, *** *p* < 0.001.

In particular, to identify new potential drugs with a synergistic effect with ruxolitinib, we exposed IL89_CL#3488 to a drug library consisting of 433 compounds mapping to ~634 targets (Table S7), at a concentration of 1 μM for 72 h, in the presence or absence of ruxolitinib (0.5 µM). A metabolic readout was used as a surrogate of cell viability. Firstly, we demonstrated a strong concordance among replicates, as depicted in the PCA in Figure 6A.

Then, we specifically looked for compounds differentially active in the presence or absence of ruxolitinib (Figure 6B). Four different drugs (17-AAG, KPT-185, ganetespib, and NH125) were statistically more active in the presence of the JAK inhibitor (Figure 6C). Two of these drugs (17-AAG and ganetespib) target the chaperone protein HSP90, whose inhibition has already been shown to have a synergistic effect with JAK/STAT inhibition in several cancers [39]. This data provides a rationale for the development of new combination approaches using ruxolitinib plus HSP90 inhibitors to be tested in vivo and in clinical trials.

To further confirm the HTS data, we also developed a new flow cytometry-based screening approach to simultaneously test 40 different compounds chosen based on their efficacy in HTS tests. Additional compounds, recently approved by the FDA or undergoing clinical trial evaluations in PTCL (chidamide, pralatrexate, idelalisib, and TGR1202), were also evaluated (Table S8). As depicted in Figure 6D, 23 of these compounds displayed statistically significant activity (compared to controls) in inducing IL89_CL#3488 cell death, providing new candidates for further pre-clinical and clinical studies.

Taken together, these results provide the proof of principle for a platform that could be readily used to identify new drug combinations in pre-clinical settings.

## 3. Discussion

BIA-ALCL was recognized as an independent entity within the 2017 revised version of the WHO classification [6]. Despite its rarity, BIA-ALCL has gained much attention from the media and regulatory agencies [40,41,42,43].

Here, we describe the first BIA-ALCL PDTX model (IL89) and its derived continuous cell line (IL89_CL#3488). Their phenotypic and genomic/transcriptomic profiles confirmed the faithful correspondence between the models and the matched tumor biopsy specimen.

The molecular features of BIA-ALCL have recently been unraveled, showing a key role of cytokines and JAK/STAT signaling in the pathogenesis of the disease [44]. Laurent et al. recently analyzed the molecular landscape of a large cohort of BIA-ALCL biopsies. Their study discovered a high degree of mutations in genes related to the epigenetic regulation of gene expression (besides the already known alteration of the JAK/STAT pathway) [9]. Of note, here, we demonstrated that the IL89 models are in line with the previously reported data. In fact, IL89 retained the specific transcriptomic signatures previously associated with BIA-ALCL [34]. Additionally, IL89 not only displayed a canonical G1097D JAK1 mutation, but also additional defects in epigenetic genes (KMT2C). There may be a particular association between BIA ALCL and JAK1 G1097D, as shown in the COSMIC database [45], but this finding needs validation in large BIA-ALCL cohorts.

Using deep sequencing, we discovered a series of putative pathogenetic alterations corresponding to both oncogenes and tumor suppressor genes, including APLK2, COL12A1, EPHA6, LHCGR, LRP1B, and RLTPR. This approach unveiled their presence, even in the primary lesion, which was in contrast with those obtained by WES. We suspect that this apparent disparity is due to a low tumor content combined with the limited sensitivity of WES in detecting low-frequency variants. To precisely define the genomic landscape of BIA-ALCL, we then took advantage of the enriched lymphoma content, enriched by the PDTX models, and the high sensitivity of deep sequencing. This latter approach can interrogate 538 genes, including those most frequently deregulated in PTCL, which can be informative regarding clonal evolution. Using bioinformatic backtracking on targeted sequences, we were able to prove that all except one mutation in the genes included in the panel could be detected in the primary lesion, suggesting that the genotype of the PDTX and cell lines was largely maintained over time. The data also suggest a certain degree of subclonal heterogeneity within the primary lesion, with low-frequency clones capable of effectively expanding/engrafting in the NSG mice and carrying, together with canonical mutations, few novel defects. Functional studies will be needed to validate the pathogenetic impact of these novel defects in BIA-ALCL. Toward this end, these discoveries may have an effect on the treatment of these lymphomas. Compounds targeting these vulnerabilities, recently described in some systemic ALCL and other T-cell lymphomas [37,46] should be tested in pre-clinical models, opening the way to the possible design of innovative trials for BIA-ALCL patients and other PTCL subtypes. PDTX are optimal platforms for carrying out pre-clinical studies. In fact, they not only faithfully resemble the primary donor samples and are stable along passages, but also grow within a rich host environment that better recapitulates the genetic landscape of the primary human disease [47]. We reported the feasibility of using the IL89 model to perform pre-clinical tests, which could foster new clinical trials. BIA-ALCL frequently carry mutations within the JAK/STAT3 pathways, but it is still unclear whether treatment targeting this pathway may have a real clinical impact. Of note, Chen and colleagues previously showed that the in vitro downregulation of JAK signaling could control growth and impair survival [48]. In the last few years, JAK/STAT inhibitors have entered into the armamentarium for the treatment of PTCL. Indeed, several reports and clinical trials have been proposed [37]. Here, as proof of principle, we tested the response to the JAK1/2 inhibitor ruxolitinib of IL89. We reasoned that, since our model retains the G1097D JAK1 alteration, we had the opportunity to investigate whether this defect could maintain the neoplastic phenotype [33]. Here, we have extended these studies and proven that JAK inhibitors can decrease the phosphorylated status of STAT3 and lead to an increased cell death rate and cell cycle arrest. The in vivo treatment with ruxolitinib led to a sharp reduction of IL89 growth, associated with a complete remission of the disease in all treated mice up to almost two months from the initial treatment. This period, however, was inevitably followed by a relapse. Considering the relatively low toxicity of some of these compounds, we envision that JAK inhibitors could be used in different settings aimed at preventing lymphoma progression. It will eventually be of interest to test whether these drugs will need to be delivered chronically, like in other scenarios (MPN) [49], to retain the neoplastic cells in dormancy, or whether treatment and lymphoma eradication will ultimately require combination with other drugs (e.g., brentuximab). Remarkably, the oncogenic addiction of the IL89_#3488_CL cell line to the STAT3 signaling pathway was maintained, even after a long period of in vitro culture in the absence of lymphokine supplementation, strongly highlighting the critical role of this signaling for the maintenance of the neoplastic phenotype and thus the therapeutic feasibility of compounds targeting STAT3.

We decided to take further advantage of the models generated in this study as an instrument for the discovery of new therapeutic regimens in high throughput settings. Of note, we demonstrated the feasibility of an HTS drug discovery using a large library of compounds in combination with ruxolitinib. The robustness and the discovery potential of our findings were further confirmed in a parallel screening in flow cytometry testing 40 drugs chosen in accordance with the HTS data and ongoing clinical trials. Interestingly, HSP90 inhibition has been proposed to act synergistically with JAK/STAT pathway blockage in IL89 BIA-ALCL. This result, appropriately validated in vitro and in vivo, represents a potential precision medicine approach to be applied in rare cases of relapsed/refractory BIA-ALCL patients with a molecular profile consistent with the IL89 model landscape.

Along the serial passages in vivo and prolonged in vitro culture, the IL89 models changed their relationship with the mouse host environment. These changes suggest that although BIA-ALCL may rely on host signals, this dependence can be overcome or bypassed; a scenario that is reminiscent of several familiar clinical habitat-changing processes, such as local invasion and distal dissemination. If this hypothesis is correct, these models may be ideal for the definition of the molecular progression of BIA-ALCL.

Collectively, we believe that this and other PDTX models will not only provide informative tools to investigate the molecular mechanisms of lymphogenesis, but will also foster new drug discoveries and the implementation of novel therapeutic strategies in genomically-annotated lymphomas.

## 4. Materials and Methods

### 4.1. Patient Sample

A naïve fresh BIA-ALCL seroma sample was obtained from the Memorial Sloan Kettering Cancer Center (MSKCC). Informed consent was obtained in accordance with the MSKCC ethical committees (IRB#: 06-107). Diagnoses were assigned according to the WHO classification by at least two experienced pathologists. Representative formalin-fixed tumor sections were processed for immunohistochemical analysis on a semi-automated strainer [50]. Pathological samples not required for diagnostic procedures were collected and placed in a sterile falcon tube. Upon arrival, patient material was centrifuged on a Ficoll Paque (Sigma-Aldrich, Darmstadt, Germany) gradient according to the manufacturer’s protocol, and isolated mononucleated cells were used for implantation in mice. Additional cells were vitally frozen in RPMI1604 media (Sigma-Aldrich, Darmstadt, Germany) supplemented with 20% FBS (Gibco, Waltham, MA, USA) and 10% DMSO (Sigma-Aldrich, Darmstadt, Germany), and stored at −80 °C for re-implantation and nucleic acid extraction.

### 4.2. PDX Establishment and Propagation

Male and female NOD.Cg-Prkdcscid Il2rgtm1Wjl/SzJ (NSG) and NOD.Cg-B2mtm1Unc Prkdcscid Il2rgtm1Wjl/SzJ (NSG beta2) mice were purchased from Jackson Laboratory (Bar Harbor, ME, USA) and bred within the Weill Cornell Medicine (WCM) Animal Resource, under strict specific and opportunistic pathogen-free (SOPF) conditions. The animal protocols were reviewed and approved by the Animal Committee of WCM (protocol 2014-0024). For seroma cell implantation, 2 × 10^6^ cells were implanted subcutaneously in Matrigel (Corning, New York, NY, USA) (1:1). For PDTX serial passaging in mice, tissue fragments (0.1 × 0.3 × 0.3 cm) were implanted in a subcutaneous (s.c.) pocket on the flank areas of mice. Before the procedure, mice were anesthetized using inhaled isofluorane (VWR, Radnor, PA, USA). The surgical site was prepared by shaving the fur and rubbing it with 70% ethanol wipes. Ocular sterile ointment (VWR, RADNOR, PA, USA) was applied to both eyes. Surgery was carried out in a sterile type 2 biological hood and fragments were delivered using TROCAR (VWR, RADNOR, PA, USA). Veterinary surgical glue (3M, Vetbond) was used to close the wound. The tumor size was evaluated twice a week using a digital caliper (VWR, RADNOR, PA, USA). Recipient animals were also regularly monitored for additional symptoms of tumoral dissemination in organs, such as heavy breathing, weight and fur loss, diarrhea, a hunched posture, reduced feeding, and dehydration. Mice were euthanized at early signs of distress in a CO_2_ chamber. Tumor grafts and organs were collected for histologic evaluation, extensive dry and vital cryopreservation (liquid nitrogen in RPMI1604 media supplemented with 20% FBS and 10% DMSO), and secondary grafting.

### 4.3. Isolation of Viable PDX-Derived Tumor Cells

Tumor tissue was finely dry minced using sterile blades and digested for 30–45 min at 37 °C. Digestion media was composed of RPMI1640 (Sigma-Aldrich, Darmstadt, Germany) and digestion buffer (4:1). The digestion buffer was prepared according to the following: 140 nM NaCl (Sigma-Aldrich, Darmstadt, Germany), 5 mM KCl (Sigma-Aldrich, Darmstadt, Germany), 2.5 mM Phosphate buffer pH7.4 (prepared by dissolving 3.1 g of NaH_2_PO_4_-H_2_O and 10.9 g of Na2HPO4 anhydrous in 1 L of sterile cell culture grade water), 10 mM Hepes (Sigma-Aldrich, Darmstadt, Germany), 2 mM CaCl_2_ (Sigma-Aldrich, Darmstadt, Germany), 1.3 mM MgCl_2_ (Sigma-Aldrich, Darmstadt, Germany), 25 mg/mL Collagenase A (Roche, Basel, Switzerland), 25 mg/mL Dispase II (Sigma-Aldrich, Darmstadt, Germany), and 250 mg/mL DNAase (Roche, Basel, Switzerland). Digested tissue was filtered through 70 μm nylon filters (Corning, New York, NY, USA) and the resulting cell suspension was washed twice with PBS (Sigma-Aldrich, Darmstadt, Germany). PDTC were resuspended in RPMI1640 (Sigma-Aldrich, Darmstadt, Germany) plus 20% FBS (Gibco, Waltham, MA, USA) and seeded at 1 million/mL in T150 flasks (Corning, New York, NY, USA) overnight. The day after, floating T-cells were separated from stromal cells attached to the flasks and centrifuged on a Ficoll Paque (Sigma-Aldrich, Darmstadt, Germany) gradient to remove dead cells, red blood cells, and debris, in order to isolate a pure (>95%) and viable (>95%) T-cell population. Stromal cells were cultured (RPMI1640 20% FBS - Sigma-Aldrich, Darmstadt, Germany) and used for experimental purposes if needed. T-cell suspensions were analyzed by flow cytometry using a panel of monoclonal antibodies against human T-cell surface markers.

### 4.4. Histopathological Analyses

Tissues were recovered 0.5–1 h after mouse sacrifice, fixed in 10% neutral buffered formalin (Sigma-Aldrich, Darmstadt, Germany), and processed for histology and immunohistochemistry. Formalin Fixed Paraffin Embedded sections were stained with hematoxylin and eosin and immunostained with monoclonal antibodies against CD30 (Leica, Buffalo Grove, IL, USA), CD3 (Thermo Scientific, Waltham, MA, USA), CD4, CD8, CD25 (Becton Dickinson, Franklin Lakes, NJ, USA), and pSTAT3 (Cell Signaling Technology, Danvers, MA, USA) using a semi-automated unit (Leica, Buffalo Grove, MA, USA). Immunohistochemistry was performed on 4 μm sections of multiple organs (lungs, spleen, liver, hearth, kidney, and tumor mass). All of the antibodies used were diluted in PBS (Sigma-Aldrich, Darmstadt, Germany).

### 4.5. Multicolor Flow Cytometry

Flow cytometry was performed by staining lymphoma cells with a mix of antibodies diluted 1:100 in PBS and recognizing T- and B-cell markers. The antibodies used are summarized in Table S1 and were purchased from BD Biosciences (Becton Dickinson, Franklin Lakes, NJ, USA). Briefly, lymphoma cells were identified after gating on human CD45+ cells, and the selected markers were analyzed inside the human CD45-positive cell population. At least 10,000 events were acquired. Samples were run on the BD FACSAria™ and analyzed with the BD FACSDiva software.

### 4.6. Identification of Clonal Antigen Receptor Gene Rearrangements

Clonal rearrangements of the T-cell receptor (TCR) genes were determined using the Invivoscribe kit (Invivoscribe Technologies, San Diego, CA, USA) based on the BIOMED-2 assay. The standardized multiplex PCR assay detects the vast majority of clonal TCR gamma 1 gene rearrangements using only two multiplex master mixes targeting conserved regions within the variable (V) and the joining (J) regions that flank the unique hypervariable antigen-binding region 3 (CDR3). PCR products were analyzed by capillary electrophoresis (CE) using the ABI 3500 Genetic Analyzer (Thermo Scientific, Waltham, MA, USA).

### 4.7. DNA and RNA Extraction

Total RNA extraction was performed starting from dry frozen tumor graft tissues using TRIZOL (Invitrogen, Thermo Scientific, Waltham, MA, USA) and the quality was checked on an Agilent Bioanalizer (Agilent Technologies, Santa Clara, CA, USA). Samples with an RNA integrity number (RIN) >7 were selected for further analysis. Genomic DNA was extracted from frozen tissues using phenol/chloroform (Sigma-Aldrich, Darmstadt, Germany). The quantity and quality were checked using the Agilent Tapestation (Agilent Technologies, Santa Clara, CA, USA) and Qubit (Invitrogen, Thermo Scientific, Waltham, MA, USA).

### 4.8. Total RNA and Whole-Exome Sequencing

Trizol-extracted total RNA was used for cDNA total library preparation using TruSeq-Stranded Total RNA sample preparation (HS protocol), following the manufacturer’s instructions (Illumina, San Diego, CA, USA). The DNA1000 Kit (Agilent Technologies, Santa Clara, CA, USA) was used to size and quantify the library preparation on an Agilent 2100 Bioanalyzer (Agilent Technologies, Santa Clara, CA, USA). Sequencing data was aligned to the human reference genome (b37) using the STAR v2.3.5 aligner [51] after human-mouse read disambiguation via BBsplit v37.76. Gene counts were calculated by using featureCounts [52] v1.4.6 with respect to Gencode v19 annotations.

For whole-exome sequencing, genomic DNA was used to prepare the libraries by employing the SureSelect 6.0 kit (Illumina, San Diego, CA, USA), according to the manufacturer’s protocol. Whole-exome sequencing data was aligned with the human reference genome (b37) with the mem algorithm from the BWA program v-0.7.12 [53]. Duplicate reads were removed using the MarkDuplicates command from Picard v1.124 (http://broadinstitute.github.io/picard/), and local realignment around indels was performed using ABRA v0.92 [54]. Somatic mutations were identified with MuTect v1.1.5. FACETS v0.9.7-13 was employed for copy number analysis [55], and the segmentation was visualized in R with the plotAberration function from the copynumber package [56]. Both RNA and DNA libraries were sequenced on Illumina HiSeq 4000 (Illumina, San Diego, CA, USA) (paired end, 100 bp or 50 bp).

### 4.9. Targeted Deep Sequencing

A targeted sequencing gene panel including coding exons and splice sites of 538 genes (target region: ~3.2 Mb) recurrently mutated (>2) in mature T-cell neoplasms, as well as genomic regions corresponding to recurrent translocations, were designed to investigate the genomic profile of the primary and PDX tumors. Using an input of genomic DNA of at least 100 ng isolated from frozen tissues, the next-generation sequencing (NGS) libraries were constructed using the KAPA Hyperplus Kit (Roche, Basel, Switzerland), and hybrid selection was performed with the Twist Library Prep Kit (Twist Biosciences, San Francisco, CA, USA), according to the manufacturer’s protocols. Multiplexed libraries were sequenced using 150-bp paired end Hiseq4000 sequencers (Illumina, San Diego, CA, USA). Commercial software (NEXTGENe, Softgenetics, State College, PA, USA) was used to perform bioinformatic analysis (SNV and INDEL variant calls) with standard settings recommended by the manufacturer. Specifically, cutoff values for the variant allele frequency, population frequencies, and strand bias were set at 5%, 0.01%, and 1:5, respectively. For PDX samples, reads originating from the mouse genome were removed by filtering out reads with two or less unmatched bases compared to the mouse reference genome (GRCm38).

### 4.10. Cell Culture and In Vitro Treatments

Patient-derived tumor cells, the IL89_CL#3488 cell line, and patient-derived stromal cells were cultured in RPMI1640 media (Sigma-Aldrich, Darmstadt, Germany) plus 20% FBS (Gibco, Waltham, MA, USA) supplemented with 100 U/mL glutammine (Sigma-Aldrich, Darmstadt, Germany), Normocin 1:500 (InVivoGen, San Diego, CA, USA), and 100 µg/mL streptomycin (Sigma-Aldrich, Darmstadt, Germany), and maintained at 37 °C in a humidified 5% CO_2_ atmosphere. Ruxolitinib (JAK1/2 inhibitor) and tofacitinib were purchased from Selleckem (Houston, TX, USA).

### 4.11. Functional Experiments

All of the experiments were performed in triplicate and repeated at least three times. The cell viability was assessed by a trypan blue exclusion count (Invitrogen, Thermo Scientific, Waltham, MA, USA), whereas the cell metabolism was evaluated using luminescence of the CTG-tagged ATP kit (cell titer glo Promega, Madison, WI, USA). Cells were stained according to the manufacturer’s protocol; after 10 min of incubation, the plates were analyzed on a plate reader (Synergy 4, BioTek, Winooski, VT, USA).

Apoptosis was detected using the Annexin V-7AAD Apoptosis Detection Kit I (BD Pharmingen, Becton Dickinson, Franklin Lakes, NJ, USA). Briefly, cells were resuspended in 100 μL of 1× binding buffer, 5 μL of 7AAD, and 5 μL of Annexin V, and then incubated for 15 min at room temperature. Apoptotic cells were analyzed by flow cytometry (BD LSR-II).

The cell cycle was analyzed via propidium iodide (PI) incorporation using the Propidium Iodide Flow Cytometry Kit (Abcam, Cambridge, UK). Cells were washed in PBS/BSA 0.5% (Sigma-Aldrich, Darmstadt, Germany) stained with PI in the presence of RNAase (1×). Following incubation at 37 °C for 15 min in the dark, nuclei were analyzed with a flow cytometer (BD LSR-II, Becton Dickinson, Franklin Lakes, NJ, USA). Cellular debris was excluded from analyses by raising the forward scatter threshold, and the DNA content of the nuclei was registered on a logarithmic scale. The percentage of elements in the hypodiploid region was calculated.

### 4.12. Protein Isolation and Western Blotting

Cells were lysed in JST buffer (Tris-HCl 20 mM Ph7.5, 150 mM NaCl, 1% Triton X-100, 5 mM EDTA, 1 mM Na_3_VO_4_, 1 mM PMSF, 10 mM NaF, and 1X protease inhibitor cocktail, Sigma-Aldrich, Darmstadt, Germany). The protein concentration was determined with the DC protein assay (Bio-Rad Laboratories, Hercules, CA, USA) using bovine serum albumin (Sigma-Aldrich, Darmstadt, Germany) as the standard, and equal amounts of protein were analyzed by SDS-PAGE (12% acrylamide). Gels were electroblotted into nitrocellulose membranes (G & E Healthcare, Chicago, IL, USA). Membranes were blocked for 1 h with 5% non-fat dry milk (Sigma-Aldrich, Darmstadt, Germany) in PBS plus 0.1% Tween-20 and incubated at 4 °C overnight with the primary antibody. Detection was performed with peroxidase-conjugated secondary antibodies, using the enhanced chemiluminescence system (Thermo Euroclone, Pero, Italy). The primary antibodies used were anti-phosphoSTAT3 and anti-STAT3 (Cell Signaling, Beverly, MA, USA).

### 4.13. Pre-Clinical In Vivo Studies

For in vivo experiments, xenografts were surgically implanted as described, in 8 and 16 week-old adult female NSG mice. Ruxolitinib (and the relative vehicle) was provided ad libitum (in feed, initial concentration 2000 mg/kg). Mice were closely followed for symptoms of tumor progression until moribund. The tumor burden was evaluated with a digital caliper twice a week, along with the body weight as a surrogate of drug toxicity.

### 4.14. High-Throughput Drug Screening (433 Drugs)

The drug-screening library, composed of 433 targeted-compounds, was purchased from Selleckchem (Houston, TX, USA), and consisted of a subset of Selleckchem’s Targeted Selective Inhibitory Library. Drugs were selected based on current clinical applications (FDA approved), their selectivity (target of canonical signaling pathways (JAK/STAT, Ras/ERK, PI3K/ATK, B-catenin, epigenetic, anti-apoptotic, etc.)), and their redundancy (multiple drugs targeting the same pathways. Collectively a total of 634 proteins were targeted. Drug screening plates were prepared at a concentration of 1 µM, spanning 2× 384-well plates using the Tecan Freedom EVO 150 (Tecan, Männedorf, Switzerland) in the High Throughout and Spectroscopy Facility at Rockefeller University. In total, ~33,000 IL89_CL#3488 cells were added per well at a 50 µL total volume (drug solution + cells) and incubated at 37 °C for 72 h. In selected samples, ruxolitinib at a final concentration of 0.5 µM was added. After drug incubation, the cell viability was evaluated based on the luminescence of CTG-tagged ATP (cell titer glo Promega, Madison, WI, USA), and assessed using a plate reader (Synergy 4, Biotek, Winooski, VT, USA), and the data was processed, analyzed, and plotted using Matlab (Mathworks, Natick, MA, USA). To determine the compound activity, each data point was normalized to its corresponding in-plate vehicle control (16 wells of vehicle controls per plate), and then linearized to transform the response-matrix (16 × 33) into a 433 × 1 drug-response vector. To assess the degree of concordance, sample replicates were plotted and analyzed using principle component analysis (PCA).

To evaluate the differences in sample responses cultured with and without ruxolitinib, we computed the differential (viability in condition 1–viability in condition 2), as previously shown in Pera et al. [57]. Briefly, the standard deviation and mean were computed across the two conditions and the ratio of the standard deviation versus the means was computed.

### 4.15. High-Throughput Drug Screening (40 Drugs)

For the 40-drug HTS, the following conditions were applied: IL89_CL#3488 with Cell Tracer Violet (1 μM, Invitrogen, Thermo Scientific, Waltham, MA, USA), washed and plated (83,000 cells/well) in 96-well plates and challenged with the drug library (1 µM) in duplicate/triplicate. After 72 h, all cells were collected and stained with propidium iodide (Sigma-Aldrich, Darmstadt, Germany). The cell viability was assessed by HTS flow cytometry (BD FACSCelesta, Becton Dickinson, Franklin Lakes, NJ, USA). At least 10,000 events were recorded per well. Flow data were analyzed by FCSExpress 6 (DeNovo Software) and Prism 8 (GraphPad).

## 5. Conclusions

An increased interest in investigating BIA-ALCL risk factors, pathogenetic mechanisms, and prognosis has emerged in recent years. Besides continuous scientific advancements, a complete understanding of the BIA-ALCL features has been hampered by the rarity of the disease and the scarcity of reliable models. Here, we report the generation and characterization of the first BIA-ALCL PDTX model and the derived continuous cell line. Our model closely recapitulates the primary corresponding lymphoma and represents a reliable platform for drug discovery. Finally, pre-clinical HTS using IL89 revealed a potential new synergic effect of HSP90 and JAK/STAT inhibitors in relapsed/refractory BIA-ALCL. We predict that the described models will finally foster the dissection of the molecular features of the BIA-ALCL and the development of new precision medicine approaches to be applied in rare cases of aggressive BIA-ALCL.

## Figures and Tables

**Table 1:** Patient’s clinical features.

Age at Diagnosis	7th Decade
Gender	F
Ethnicity	White
Risk factors	Previous smoker
Other neoplasms	Breast cancer
Refractory to prior treatments	No
IHC	
positive markers	CD30+, CD4+, granzyme B+
negative markers	ALK−, TIA-1−, CD3−, CD20−
Ann Arbor stage	0–2
LDH	normal (232, max: 246)
ECOG Performance status	1–2
IPI	1
Extranodal sites	1
B symptoms	No
Bulky mass (>10 cm)	No
Spleen involvement	No
Peripheral blood involvement	No
Karnofsky Performance Status (KPS)	90
Therapy after diagnosis	Surgical removal of implants and capsule
Radiotherapy	No
Bone marrow transplant	No
Best clinical response	CR
Progression/relapse	No
Status	Censured

## Supplementary Materials

The following are available online at https://www.mdpi.com/2072-6694/12/6/1603/s1: Figure S1: Additional IL89 phenotypical characterization data referred to in Figure 1, Figure S2: validation of the mutation found in ultra-deep sequencing, Figure S3: Uncropped Western Blot data and weight of the mice referred to in Figure 4, Figure S4: Additional IL89_CL#3488 phenotypical characterization and sensitivity to ruxolitinib referred to in Figure 5, Table S1: Flow cytometry markers on IL89 PDTX, Table S2: Mutational landscape of IL89 PDTX, Table S3: Chromosomal distribution of mutations of IL89 PDTX, Table S4: Main oncogenic genes identified as mutated in IL89 BIA-ALCL, Table S5: List of genes and exons covered by targeted deep sequencing, Table S6: Cell line vs. PDTX passage T5 flow cytometry markers, Table S7: The 433 drugs used for HTS, Table S8: The 40 drugs used for flow cytometry-based HTS.
