# Supplementary material for: A Novel JAK1 Mutant Breast Implant-Associated Anaplastic Large Cell Lymphoma Patient-Derived Xenograft Fostering Pre-Clinical Discoveries
Source: Cancers (Basel). 2020 Jun 17;12(6):1603. doi: 10.3390/cancers12061603 (PMC7352499; doi:10.3390/cancers12061603)
Supplement: Supplementary file 1 [file cancers-12-01603-s001.pdf]

Supplementary Materials: A Novel JAK1 Mutant Breast Implant-Associated Anaplastic Large Cell Lymphoma Patient-Derived Xenograft Fostering Pre-Clinical Discoveries

Danilo Fiore, Luca Vincenzo Cappelli, Paul Zumbo, Jude M. Phillip, Zhaoqi Liu, Shuhua Cheng, Liron Yoffe, Paola Ghione, Federica Di Maggio, Ahmet Dogan, Inna Khodos, Elisa de Stanchina, Joseph Casano, Clarisse Kayembe, Wayne Tam, Doron Betel, Robin Foa', Leandro Cerchietti, Raul Rabadan, Steven Horwitz, David M. Weinstock and Giorgio Inghirami

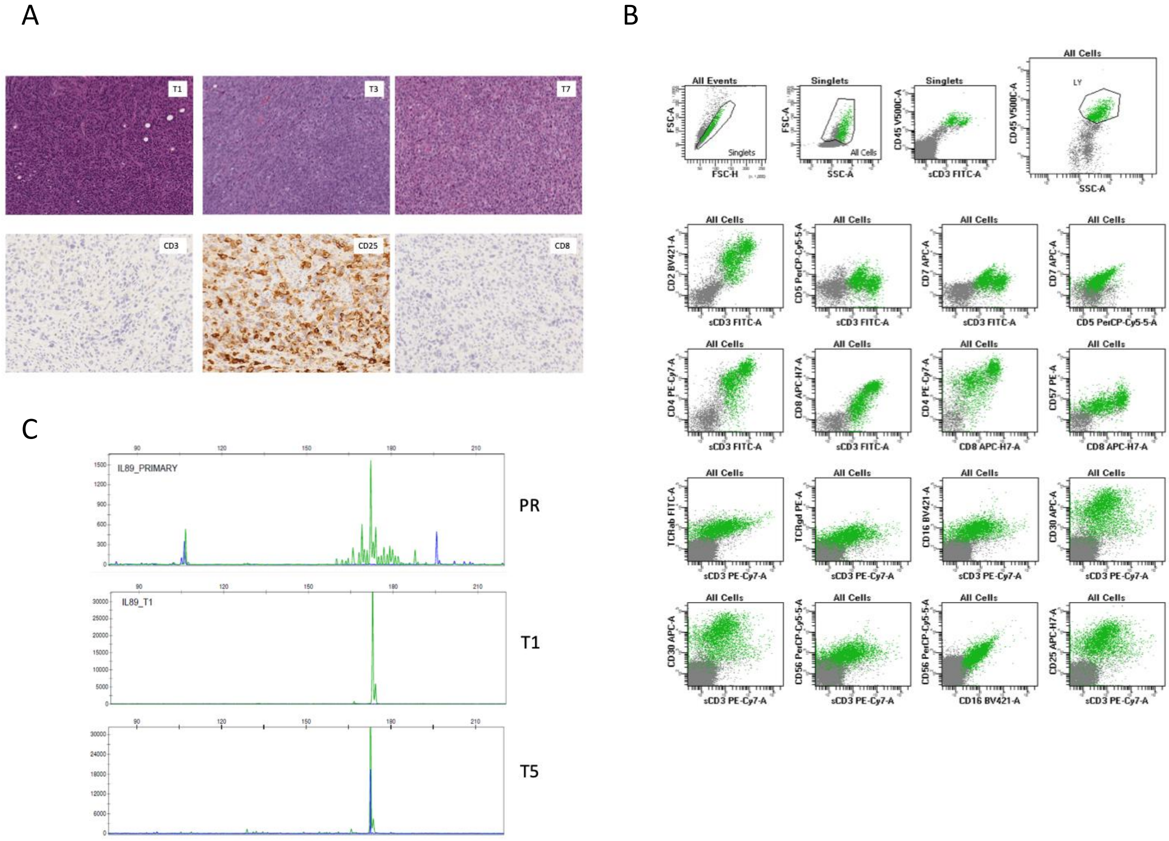

Figure S1. (A) Histology micrografts on IL89 PDTX show overall similarity between T1 T3 and T7 passages (upper panels). Immunohistochemical stains with the indicated antibodies (anti-CD3, anti-CD25 and anti-CD8 [x20]) (lower panels). (B) Flow cytometry panel comprehensive of the most represented surface T-cell lymphoma markers, including: CD2, CD3, CD4, CD5, CD8, CD16, CD25, CD30, CD56, TCRab, TCRgd. IL89 PDTX passage T3 is here depicted for illustration purposes. (C) Analysis of the TCR gamma specific rearrangement clonality in IL89 diagnostic sample and correspondent PDTX after 1 and 5 passages (T1 and T5).

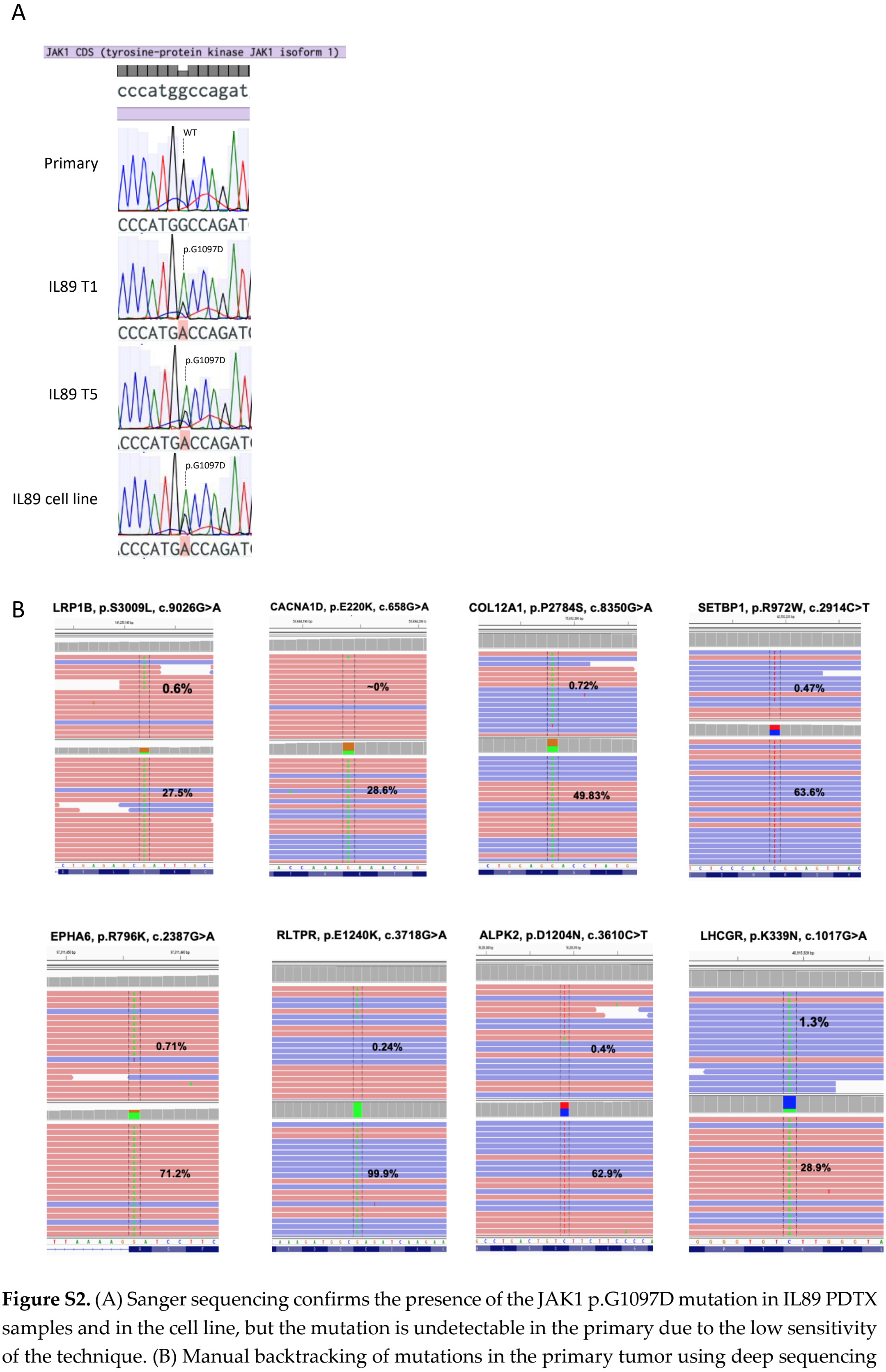

Figure S2. (A) Sanger sequencing confirms the presence of the JAK1 p.G1097D mutation in IL89 PDTX samples and in the cell line, but the mutation is undetectable in the primary due to the low sensitivity of the technique. (B) Manual backtracking of mutations in the primary tumor using deep sequencing data allowed for the identification of several hits at a very low VAF compared to the PDTX-T5.

A

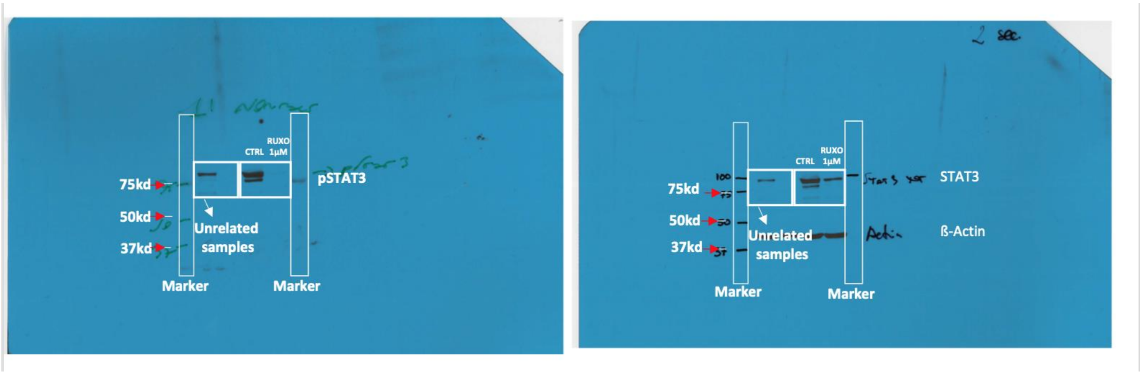

B

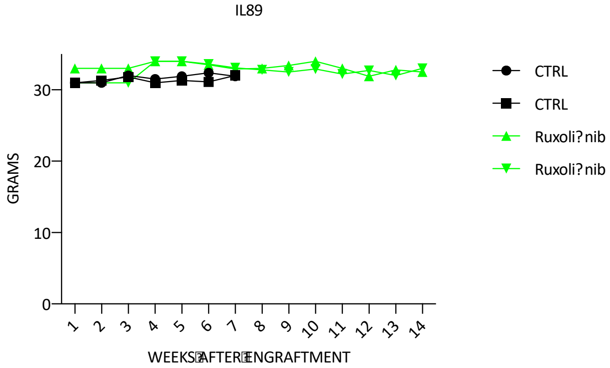

Figure S3. Additional data relative to Figure 4. (A) Uncropped film pictures relative to the WB in Figure 4A. (B) Weight of mice treated with ruxolitinib vs control. Mice who received ruxolitinib did not show any significant toxicity as demonstrated by the substantially constant weight along the treatment course.

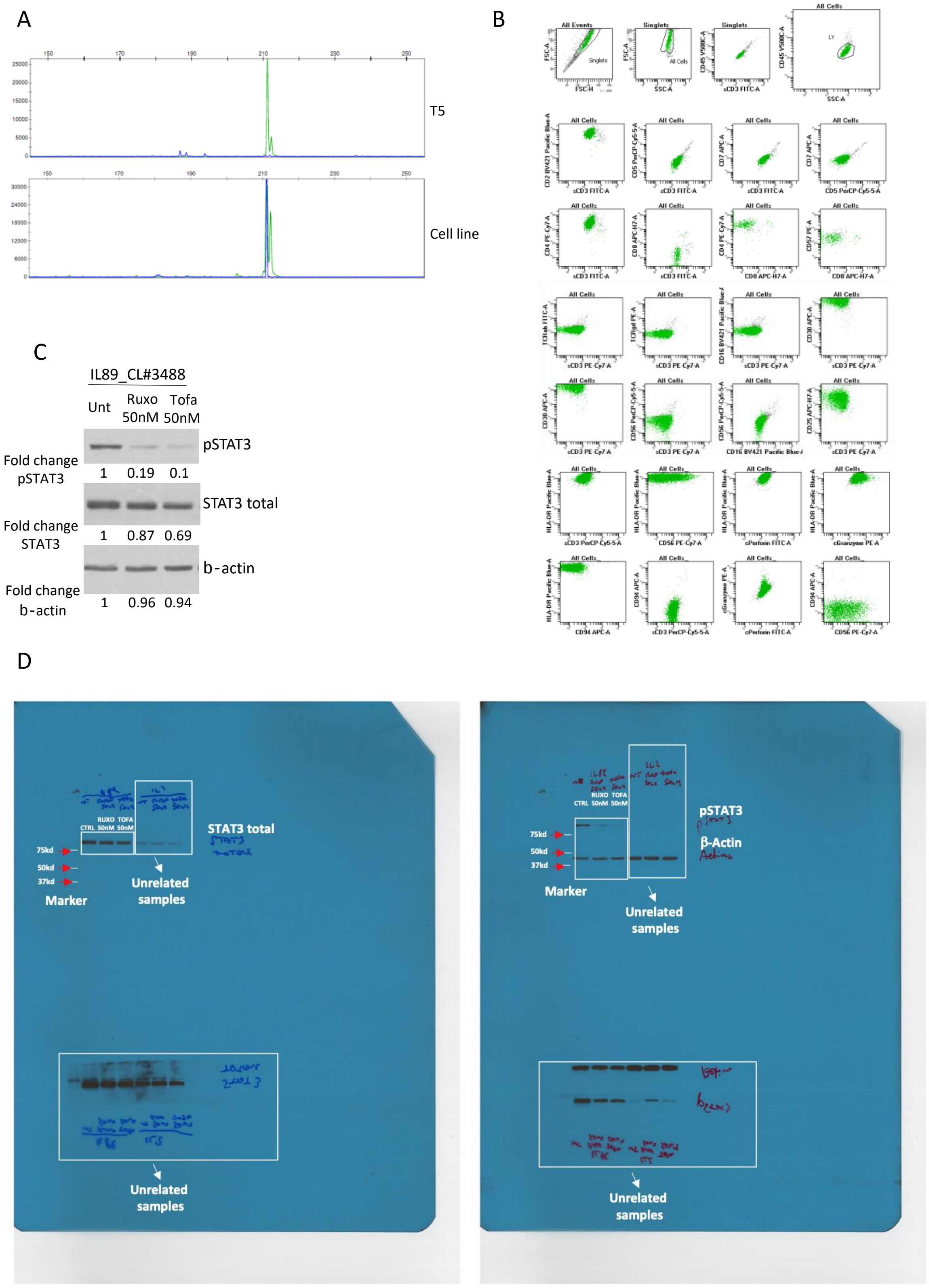
A
T5
Cell line
B
C
IL89_CL#3488
Unt
Ruxo 50nM
Tofa 50nM
pSTAT3
Fold change pSTAT3
1
0.19
0.1
STAT3 total
Fold change STAT3
1
0.87
0.69
b-actin
Fold change b-actin
1
0.96
0.94
D
STAT3 total
75kd
50kd
37kd
Marker
Unrelated samples
CTRL
RUXO 50nM
TOFA 50nM
pSTAT3
β-Actin
Unrelated samples
Marker
Unrelated samples
Unrelated samples

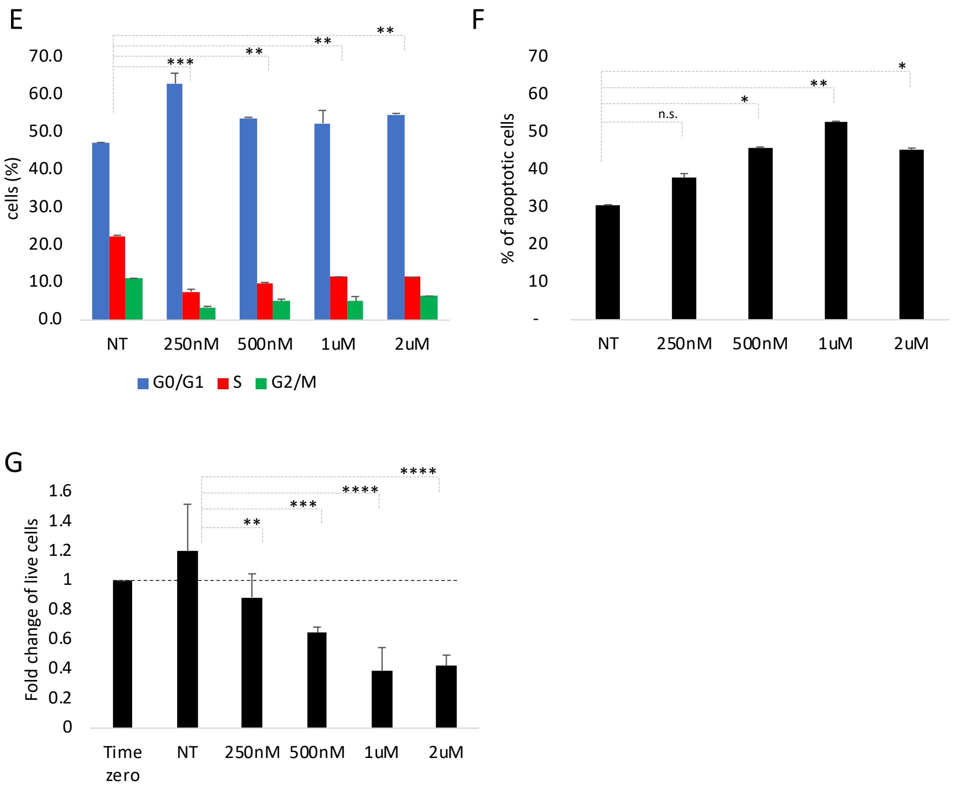

Figure S4. (A) Analysis of the TCR gamma specific rearrangement clonality in IL89 passage T5 and IL89_CL#3488. (B) Flow cytometry on IL89_CL#3488 with a panel comprehensive of the most represented surface T-cell lymphoma markers, including: CD2, CD3, CD4, CD5, CD8, CD16, CD25, CD30, CD56, TCRab, TCRgd. (C) Western Blot analysis in IL89_CL#3488 treated with 50nM of ruxolitinib or 50 nM tofacitinib for 24hr. (D) Uncropped film pictures relative to the WB in Figure Supplemental fig S4C. (E-F-G) Ruxolitinib treatment (different concentrations, 72hr) in IL89_CL#3488 resulted into a cell cycle arrest (E), an increase of the apoptotic rate (F), and a reduction of cell number (G) in vitro.

Table S1. flow cytometry markers on IL89 PDTX.

**Table S1: flow cytometry markers on IL89 PDTX**			
	**T1**	**T3**	**T5**
CD45	+	+	+
CD2	+	+	+
sCD3	+	+/-	+/-
CD5	-	-	-
CD7	-	-	-
CD4	+	+	+
CD8	-	-	-
CD57	-	-	-
TCRab	+	+/-	+/-
TCRgd	-	-	-
CD16	-	-	-
CD30	+	+	+
CD56	-	-	-
CD25	+/-	+/-	+/-
CD19	-	-	-
CD20	-	-	-
CD10	-	-	-
CD22	-	-	-
TdT	-	-	-

Granzyme
Perforin
CD94

Table S2. Mutational landscape of IL89 PDTX.

**Table S2: Mutational landscape of IL89 PDTX**	
**Frame-shift**	15
**In-frame insertions/deletions**	13
**Missense**	624
**Non-sense**	62
**Non-stop**	1
**Splice-site**	19
Total	734

Table S3. Chromosomal distribution of mutations of IL89 PDTX.

**Chromosome**	**mutations (n)**
1	75
2	52
3	45
4	35
5	41
6	36
7	38
8	43
9	33
10	26
11	53
12	32
13	16
14	33
15	34
16	21
17	35
18	12
19	8
20	11
21	3
22	6
X	41

Table S4. Main oncogenic genes identified as mutated in IL89 BIA-ALCL.

**Table S4: Main oncogenic genes identified as mutated in IL89 BIA-ALCL**			
**Gene**	**Function**	**Tumor**	**PMID**
**ALPK2**	Protein kinase that recognizes phosphorylation sites in which the surrounding peptides have an alpha-helical conformation (PubMed:10021370).	Colorectal adenoma	22641666
**COL12A1**	This gene encodes the alpha chain of type XII collagen, a member of the FACIT (fibril-associated collagens with interrupted triple helices) collagen family. Type XII collagen is a homotrimer found in association with type I collagen, an association that is thought to modify the interactions between collagen I fibrils and the surrounding matrix	Gastric cancer	31432110
**EPHA6**	Eph receptors are the largest family of receptor tyrosine kinases (RTKs) and are divided into two subclasses, EphA	Prostate cancer	26041887

	and EphB. Originally identified as mediators of axon guidance, Eph receptors are implicated in many processes, particularly cancer development and progression.		
**GPR110**	Probable G-protein coupled receptor 110 is a protein that in humans is encoded by the GPR110 gene. This gene encodes a member of the adhesion-GPCR receptor family	Lung, Prostate cancer	20149256
**IL22RA1**	Component of the receptor for IL20, IL22 and IL24. Component of IL22 receptor formed by IL22RA1 and IL10RB enabling IL22 signaling via JAK/STAT pathways. IL22 also induces activation of MAPK1/MAPK3 and Akt kinases pathways. Component of one of the receptor for IL20 and IL24 formed by IL22RA1 and IL20RB also signaling through STATs activation. Mediates IL24 antiangiogenic activity as well as IL24 inhibitory effect on endothelial cell tube formation and differentiation.	Pancreatic cancer	29572224
**KIAA0368**	Adapter/scaffolding protein that binds to the 26S proteasome, motor proteins and other compartment specific proteins. May couple the proteasome to different compartments including endosome, endoplasmic reticulum and centrosome. May play a role in ERAD and other enhanced proteolysis (PubMed:15496406). Promotes proteasome dissociation under oxidative stress (By similarity)	Metastatic breast	29867227
**LHCGR**	Receptor for lutropin-choriogonadotropic hormone (PubMed:11847099). The activity of this receptor is mediated by G proteins which activate adenylate cyclase (PubMed:11847099)	Ovarian cancer	26530886
**LRP1B**	LRP1B is a putative tumor suppressor and a member of the low-density lipoprotein (LDL) receptor family. The LDL receptor family have roles related to clearance of extracellular ligand and are proposed to be involved in extracellular signal transduction. silencing and down-expression of LRP1B as been observed in renal cell carcinoma and thyroid cancer. Further Deletion of LRP1B has been associated with chemotherapy resistance in high-grade serous cancers.	Melanoma, Non-small cell lung cancer (NSCLC), and others	31164891
**MMP2**	This gene is a member of the matrix metalloproteinase (MMP) gene family, that are zinc-dependent enzymes capable of cleaving components of the extracellular matrix and molecules involved in signal transduction. The protein encoded by this gene is a gelatinase A, type IV collagenase, that contains three fibronectin type II repeats in its catalytic site that allow binding of denatured type IV and V collagen and elastin. This protein is thought to be involved in multiple pathways including roles in the nervous system, endometrial menstrual breakdown, regulation of vascularization, and metastasis.	Breast cancer	29113219
**PDGFB**	This gene encodes a member of the protein family comprised of both platelet-derived growth factors (PDGF) and vascular endothelial growth factors (VEGF). The encoded preproprotein is proteolytically processed to generate platelet-derived growth factor subunit B, which can homodimerize, or alternatively, heterodimerize with the related platelet-derived growth factor subunit A. These proteins bind and activate PDGF receptor tyrosine kinases, which play a role in a wide range of developmental processes.	Melanoma, Lung, Glioblastoma, Bladder, Prostate, Colorectal and Ovarian cancers	26153649
**PDGFRA**	This gene encodes a cell surface tyrosine kinase receptor for members of the platelet-derived growth factor family. These	Gastrointestinal stromal cancer, Adenocarcinoma,	28572459

	growth factors are mitogens for cells of mesenchymal origin. The identity of the growth factor bound to a receptor monomer determines whether the functional receptor is a homodimer or a heterodimer, composed of both platelet-derived growth factor receptor alpha and beta polypeptides. Studies suggest that this gene plays a role in organ development, wound healing, and tumor progression	Glioblastoma multiforme, Colon adenocarcinoma, Melanoma	
**PRMT3**	This gene belongs to the protein arginine methyltransferase (PRMT) family. The encoded enzyme catalyzes the methylation of guanidino nitrogens of arginyl residues of proteins. The enzyme acts on 40S ribosomal protein S2 (rpS2), which is its major in-vivo substrate, and is involved in the proper maturation of the 80S ribosome. Alternative splicing results in multiple transcript variants.	Pancreatic cancer	31324208
**PTPRQ**	This locus encodes a member of the type III receptor-like protein-tyrosine phosphatase family. The encoded protein catalyzes the dephosphorylation of phosphotyrosine and phosphatidylinositol and plays roles in cellular proliferation and differentiation.	Colorectal cancer	26851024
**RLTPR**	This gene encodes a member of the CARMIL (capping protein, Arp2/3, myosin-I linker) family of proteins. The encoded protein interacts with and negatively regulates the heterodimeric capping protein and promotes cell migration.	Cutaneous T-cell lymphoma	28694326
**SETBP1**	This gene encodes a protein which contains a several motifs including a ski homology region and a SET-binding region in addition to three nuclear localization signals. The encoded protein has been shown to bind the SET nuclear oncogene which is involved in DNA replication.	Myeloid malignancies	23832012
**SOX4**	This intronless gene encodes a member of the SOX (SRY-related HMG-box) family of transcription factors involved in the regulation of embryonic development and in the determination of the cell fate. The encoded protein may act as a transcriptional regulator after forming a protein complex with other proteins, such as syndecan binding protein (syntenin). The protein may function in the apoptosis pathway leading to cell death as well as to tumorigenesis.	Epithelial cancers	28780934

Table S5. List of genes and exons covered by targeted deep sequencing.

Table S5: List of genes and exons covered by targeted deep sequencing							
AASDH	CD300LB	DNAH7	HOXA6	MLF1	PCDHA12	RFXAP	TENM1
ABCA7	CD53	DNM3	HOXA9	MLH1	PCDHGA11	RHOA	TET1
ABCC4	CD58	DNMT3A	HUWE1	MLL	PCDHGA9	RLTPR	TET2
ACSL3	CDH10	DOCK3	IDH2	MLL2	PCLO	RNF213	TET3
ACSL6	CDH11	DOCK7	IFNAR2	MLL3	PCMTD1	ROBO4	THSD7A
ACVR1C	CDH16	DOCK9	IGSF22	MOCOS	PCSK5	ROM1	TLR3
ADAM28	CDH18	DPY19L2	IL2RG	MPRIP	PDCD1	ROS1	TLX1
ADAM33	CDH19	DSPP	ING1	MSH6	PDE4DIP	RPS24	TMEM51
ADAMTS12	CDHR4	DST	INO80	MTERFD3	PDGFRB	RUNX1T1	TMPRSS2
ADAMTS5	CDKN2A	DTHD1	INSR	MTMR8	PDPK1	RXFP2	TNFAIP3
ADAMTS9	CDON	DYNC2H1	INTS8	MUC12	PEG3	RYR2	TNFRSF14
AFF3	CHD4	E2F1	IPO9	MUC16	PER1	RYR3	TNFRSF19
AKT1	CHD8	EBF1	IQCJ-SCHIP1	MUC17	PHACTR1	SACS	TNFRSF1B
ALDH1A2	CHEK2	EBF2	IRS2	MUC2	PHIP	SAMD9	TNS1
ALK	CHL1	EIF3A	JAK1	MUC5B	PIK3CB	SCAF1	TP53
ALMS1	CHST6	ENAM	JAK2	MUM1	PIK3CD	SCN1A	TPR
ALPK2	CHST7	ENO4	JAK3	MX2	PIK3R1	SCN2A	TRAF5
ALPK3	CHSY3	ENPP3	JPH3	MYBPH	PKD2L1	SDK2	TRAF6

ALPP	CIITA	EP300	KAT6A	MYC	PKHD1	SEMA3A	TRIM3
ANAPC2	CIZ1	EPB41L3	KAT6B	MYH11	PLCG1	SETBP1	TRMT12
ANK3	CLEC14A	EPC2	KCNH8	MYH7	PLCG2	SETD2	TRPA1
ANKLE1	CLK2	EPHA5	KCNK1	MYH9	PLK2	SETX	TRPM6
ANKRD50	CLTC	EPHA6	KCNN3	MYL2	PLK3	SFSWAP	TRRAP
ANO3	CMYA5	ERBB4	KCTD8	MYO18B	PLXNC1	SGK1	TSKU
APC	CNOT4	ETV1	KDM4C	MYO3A	PML	SH2B3	TTC28
ARFGEF1	CNTN3	ETV6	KDM6A	MYOD1	PMS1	SH3BP4	TTN
ARHGEF11	CNTN4	EXT2	KDR	MYT1L	POF1B	SLC13A5	TUBGCP6
ARHGEF17	CNTRL	EZH2	KIAA0922	NAA11	POLE	SLC16A14	TYK2
ARHGEF3	COL11A1	EZR	KIAA1324	NAV2	POT1	SLC17A6	TYRP1
ARID1A	COL12A1	FAM22G	KIF26A	NAV3	PPP1R9A	SLC25A24	UBR5
ARID1B	COL19A1	FAM47A	KIF5B	NCKAP5	PRDM1	SLC3A2	UGT3A1
ARID2	COL22A1	FAM71C	KIF7	NCOA1	PREX2	SLC44A5	ULK4
ARNT2	COL4A2	FAS	KIT	NCOA2	PRKCDBP	SLC6A2	UNC79
ARPP21	COL5A2	FAT1	KLHL40	NCOR1	PRKCQ	SLC8A3	UNC80
ATM	COL6A3	FAT2	KLK2	NCOR2	PRKD2	SLITRK3	USF2
ATP1A3	COL6A6	FAT3	KRAS	NEDD4	PRKG1	SMAD3	USP51
ATP2A2	CPXM2	FAT4	KRTAP1-3	NEFH	PROX1	SMARCA2	USP8
ATXN3	CRAMP1L	FBN1	KRTAP26-1	NEIL3	PRRC2C	SMARCAD1	VAV1
B2M	CREB3L1	FBN3	KTN1	NF1	PTCHD2	SMARCB1	VPS13A
BANK1	CREBBP	FBXO11	LAMA1	NFKB1	PTCHD4	SMCHD1	WDFY3
BCL9	CRTAC1	FBXW7	LAMA2	NFKB2	PTEN	SOCS1	WDFY4
BCOR	CSMD1	FEZF1	LAMC2	NIN	PTK6	SOWAHA	WDR17
BCORL1	CSMD2	FGFR1	LAT	NLRP12	PTPN1	SPAG17	WDR60
BCR	CSMD3	FH	LCK	NLRP4	PTPN13	SPATA31D1	WIF1
BIRC6	CSNK1A1	FLG	LHCGR	NLRP7	PTPN23	SPECC1	WNT7B
BMPER	CTCF	FLT3	LIFR	NOP2	PTPRB	SPEG	XIRP2
BRAF	CTNNA2	FN1	LILRB1	NOTCH1	PTPRC	SPINK5	XRCC6BP1
BRCA2	CTNNB1	FNBP1	LMF1	NOTCH2NL	PTPRD	SPTA1	ZAP70
BRIP1	CTNND1	FNBP4	LMO2	NOTCH3	PTPRF	SPTBN1	ZEB1
BRPF3	CTTNBP2	FNDC1	LOC283710	NPAT	PTPRM	SRGAP3	ZFHX3
BTBD11	CUL9	FOXA3	LOC554223	NPHS1	PTPRN2	SRRM2	ZIC4
C10orf120	CUX1	FOXO1	LPHN3	NR5A2	PTPRT	ST18	ZNF226
CACNA1C	CYYR1	FUBP3	LRP1B	NRAS	PTPRZ1	STAB1	ZNF292
CACNA1D	DDI1	FYN	LRP6	NRG1	PWWP2A	STARD9	ZNF365
CACNA1S	DDX11	GABRE	LRRC14B	NRG3	RAB9B	STAT3	ZNF462
CACNA2D1	DDX3X	GABRR1	LRRK1	NT5C3	RAD21	STAT5B	ZNF572
CALR	DENND2A	GNAI2	LSR	NTRK1	RALGAPB	STIL	ZNF579
CARD11	DHX15	GPAT2	LTBP1	NTRK3	RARA	STK11IP	ZNF608
CARS	DLC1	GPI	LYN	NUMA1	RASA2	STXBP3	ZP4
CASC5	DLGAP2	GPR27	MAGEC1	OSBPL6	RASAL2	SYNE1	ZSWIM4
CASP3	DLGAP3	GRID2	MAGI1	P2RY6	RB1	SYNPO2L	ZZEF1
CASP8AP2	DMBT1	GRIK4	MAML2	PAPLN	RBBP8	TACC2	
CBFA2T3	DMD	GTF2I	MAPK3	PAPPA2	RBFOX1	TAL1	
CCDC91	DMKN	HIST1H1B	MDN1	PASD1	RBMXL2	TATDN2	
CCR4	DMXL2	HIST1H3J	MET	PASK	RBP3	TBC1D8B	
CCR8	DNAH2	HLA-DPB1	MGAT4C	PBRM1	RECQL4	TCF12	
CD28	DNAH5	HMCN1	MICAL3	PCDH17	RELN	TCF20	
CD300C	DNAH6	HOXA2	MID1	PCDHA11	RFX4	TCF7L2	

Table S6. Cell line vs PDTX passage T5 flow cytometry markers.

**Table S6: Cell line vs PDTX passage T5 flow cytometry markers**		
	**T5**	**Cell line**
CD45	+	+
CD2	+	+
sCD3	+/-	+/-

CD5	-	-
CD7	-	-
CD4	+	+
CD8	-	-
CD57	-	-
TCRab	+/-	+/-
TCRgd	-	-
CD16	-	-
CD30	+	+
CD56	-	-
CD25	+/-	+/-
CD19	-	-
CD20	-	-
CD10	-	-
CD22	-	-
TdT	-	-
Granzyme		+/-
Perforin		-
CD94		-
HLA-DR		+

Table S7. 433 drugs used for HTS.

Table S7: 433 drugs used for HTS	
**Compounds**	**Targets**
FG-4592	EPAS1, EPO
Rizatriptan Benzoate	HTR1A
Fulvestrant	ESR
Tolfenamic Acid	PTGS2
Ramelteon	MTNR1A, MTNR1B
Trospium chloride	CHRM1
Granisetron HCl	HTR3A
A-769662	PRKAA1
Losartan Potassium (DuP 753)	AGTR
Tolazoline HCl	ADRA1A
Tenofovir Disoproxil Fumarate	antiretroviral
Ataluren (PTC124)	CFTR
Candesartan	AGTR
Belinostat (PXD101)	HDAC
Semagacestat (LY450139)	APP, NOTCH
NVP-ADW742	IGF1R
NSC 319726	TP53 (R175)
Icotinib	EGFR
Ilomastat (GM6001, Galardin)	MMP1, MMP2, MMP3, MMP7, MMP8, MMP9, MMP12, MMP14, MMP26
Rivaroxaban	F10
STF-118804	NAMPT
Rimonabant	CNR1
FLI-06	NOTCH
Sitaxentan sodium	EDNRA
ABT-263 (Navitoclax)	BCL2, BCL2L1, BCL2L2
Brinzolamide	CA2
Nilotinib (AMN-107)	ABL1
Tranylcypromine (2-PCPA) HCl	MAO, CYP2A6
Tandutinib (MLN518)	FLT3, PDGFR KIT
Zebularine	DNMT1, DNMT3A, DNMT3B
MLN8054	AURKA

PR-619	ATXN7L3, BAP1, OTUB1, OTUD1, OTUD6A, OTUDB7A, UBB, UCHL3, USP1, USP2, USP4, USP5, USP7, USP8, USP14, USP15, USP17, USP26, USP30, USP36, YOD1
Roxatidine Acetate HCl	HRH2
GSK1904529A	IGF1R, INSR
Atorvastatin Calcium	HMGCR
SNS-032 (BMS-387032)	CDK2
Naltrexone HCl	OPRK1, OPRM1, OPRD1
Ganetespib (STA-9090)	HSP90
CGS 21680 HCl	ADORA2A
Vemurafenib (PLX4032, RG7204)	BRAF (V600E)
Pancuronium dibromide	nicotinic acetylcholine receptor antagonist
Loratadine	HRH1
PNU-120596	nAChR
Ruxolitinib (INCB018424)	JAK1, JAK2
GW3965 HCl	NR1H3, NR1H2
AZD6482	PIK3CB
SB705498	TRPV1
Safinamide Mesylate	MAOB, MAOA
Tenofovir	antiretroviral
P22077	USP7, USP47
Aloxistatin	cysteine protease inhibitor
NSC697923	UBE2
Apixaban	F10
ML347	ACVR1, ACVRL1
Irinotecan HCl Trihydrate	TOP1
SSR128129E	FGFR1
VX-765	CASP1
Ferrostatin-1 (Fer-1)	VDAC
Rotundine	DRD1
MM-102	KANSL1
PF-3845	FAAH
OTX015	BRD2, BRD3, BRD4
Ibrutinib (PCI-32765)	BTK
4E1RCat	EIF4E
Tofacitinib (CP-690550,Tasocitinib)	JAK3
PHA-793887	CDK2, CDK5, CDK7
WZ811	CXCR4
Allopurinol	HCRTR1, HCRTR2
HC-030031	TRPA1
Telmisartan	AGTR
Mozavaptan	AVPR1, AVPR2
Cyproterone Acetate	AR
Sodium 4-Aminosalicylate	NFKB
GSK1292263	GPR119
SGC 0946	DOT1L
LY2157299	TGFBR1
IPA-3	PAK1
Esomeprazole Sodium	ATP4A
DMH1	ACVR1
Ozagrel	TBXA2R
AZD4547	FGFR1, FGFR2, FGFR3
GW9508	FFAR1, FFAR4
VE-821	ATR
NSC 405020	MMP14
GNF-2	ABL1
TPCA-1	IKBKB
T0901317	NR1H3, NR1H2, NR1H4

PD0325901	MAP2K1, MAP2K2
PYR-41	UBA1
U0126-EtOH	MAP2K1, MAP2K2
LDN-212854	ACVR1, ACVRL1
Ki16425	LPAR1, LPAR2, LPAR3
C646	EP300
Oxcarbazepine	SCN
Mdivi-1	DRP1, DNM1
MRS 2578	P2RY6
AGI-5198	IDH1
LY2603618	CHEK1
BTB06584	ATP5A1
NPS-2143	CASR
Veliparib (ABT-888)	PARP1, PARP2
Dalcetrapib (JTT-705, RO4607381)	CETP
Vandetanib (ZD6474)	KDR
Istradefylline	ADORA2A
Iniparib (BSI-201)	PARP1
Dabrafenib (GSK2118436)	BRAF (V600)
Finasteride	SRD5A2
Tyrphostin AG 879	ERBB2
Cilomilast	PDE4
TAE226 (NVP-TAE226)	PTK2, PTK2B
Ozagrel HCl	TBXAS1
Vildagliptin (LAF-237)	DPP4
Dynasore	DNM1, DNM2
Piceatannol	SYK
Quizartinib (AC220)	FLT3
Tenovin-6	TP53, SIRT2, SIRT1, SIRT3
Enzastaurin (LY317615)	PRKCB, PRKCA, PRKCG, PRKCE
CGP 57380	MKNK1
Bisoprolol fumarate	ADRB1
Bosutinib (SKI-606)	SRC, ABL1
S3I-201	STAT3
HA14-1	BCL2
TG100-115	PIK3CG, PIK3CD
ADL5859 HCl	OPRK1, OPRM1
Voriconazole	CYP51A1
BIBR 1532	TERT
Thiazovivin	ROCK1, ROCK2
Anastrozole	CYP19A1
SB743921	kinesin spindle protein (KSP)
EUK 134	SOD1
Bergenin	trihydroxybenzoic acid glycoside
SN-38	TOP1
CP-91149	PYGL, PYGM, PYGB
Wnt-C59 (C59)	WNT3A
NU7026	PRKDC
BAM7	BAX
ZM 306416	FLT1
(+)-JQ1	BRD4
GW9662	PPARG
KPT-185	XPO1
Pifithrin-μ	TP53, HSPBP1
Batimastat (BB-94)	MMP1, MMP2, MMP3, MMP7, MMP9
Sertraline HCl	5-HT antagonist
OG-L002	KDM1A
MK-1775	WEE1
Costunolide	TERT

AT101	BCL2, BCL2L1, MCL1
GSK690693	AKT1, AKT2, AKT3
Tropicamide	CHRM4
BMS-707035	HIV-I integrase (IN)
Raltegravir (MK-0518)	integrase (IN)
EX 527 (Selisistat)	SIRT1
CCT128930	AKT2
Pomalidomide	TNF
AS-252424	PIK3CG
Tie2 kinase inhibitor	TEK
Ouabain	ATP1B
Ranitidine	HRH2
SKI II	S1PR
Fluvastatin Sodium	HMGCR
Propranolol HCl	ADRB1
Erastin	VDAC
Ifenprodil Tartrate	GRIN
KPT-276	XPO1
AZD2461	PARP
KPT-330	XPO1
AGI-6780	IDH2
SGI-1027	DNMT1, DNMT3A, DNMT3B
Atglistatin	PNPLA2
Suvorexant (MK-4305)	HCRTR1, HCRTR2
SRT1720	SIRT1
4EGI-1	EIF4E
Exemestane	CYP19A1
NSC 23766	RAC
2-Methoxyestradiol (2-MeOE2)	HIF1A
Palbociclib (PD-0332991) HCl	CDK4, CDK6
EHop-016	RAC1, RAC3
PF-573228	PTK2
ABT-199 (GDC-0199)	BCL2
Memantine HCl	CYP2B6
PTC-209	BMI1
Trimebutine	OPRK1, OPRM1, OPRD1
CK-636	ARPC2, ARPC3
SGI-1776 free base	PIM1, FLT3, GSG2
AZD7545	PDK1, PDK2
URB597	FAAH
GW0742	PPARD
TAK-875	FFAR1
Pacritinib (SB1518)	JAK2, FLT3
KX2-391	SRC
PluriSIn #1 (NSC 14613)	SCD
Crenolanib (CP-868596)	PDGFRA, PDGFRB, FLT3 (D842V)
Enzalutamide (MDV3100)	AR
PFI-1 (PF-6405761)	BRD4
Dapagliflozin	SLC5A2
Maraviroc	CCR5
Nebivolol	ADRB1
I-BET151 (GSK1210151A)	BRD2, BRD3, BRD4
VX-809 (Lumacaftor)	CFTR
Apoptosis Activator 2	CASP3
Naproxen	PTGS1, PTGS2
Bosentan Hydrate	EDNRA, EDNRB
Acadesine	PRKAA1
E-64	CTSK
Captopril	ACE

Selumetinib (AZD6244)	MAP2K1, MAPK3, MAPK1
Tolvaptan	AVPR2
PD184352 (CI-1040)	MAP2K1, MAP2K2
OSI-906 (Linsitinib)	IGF1R, INSR
Canagliflozin	SLC5A2
CP-673451	PDGFRA, PDGFRB
Sirtinol	SIRT1, SIRT2
Methotrexate	DHFR
SAR131675	FLT4
Pralatrexate	DHFR
BML-190	CNR2
TWS119	GSK3B
IKK-16 (IKK Inhibitor VII)	IKBKB, CHUK
Enalaprilat Dihydrate	ACE
Triamterene	SCN
Clemastine Fumarate	HRH1
Fingolimod (FTY720) HCl	S1PR
Amlodipine	CACNA1C
PP2	SRC
CHIR-124	CHEK1
Temsirolimus (CCI-779, NSC 683864)	MTOR
YO-01027	APP, APPL1, NOTCH
Trichostatin A (TSA)	ALL HDACS (Except HDAC8)
PAC-1	CASP3
PHA-665752	MET
VE-822	ATR
SB203580	MAPK11, MAPK12, MAPK13, MAPK14
EPZ-6438	EZH2
KU-55933 (ATM Kinase Inhibitor)	ATM
CGK 733	ATM, ATR
WZ4002	EGFR
WZ4003	NUAK1, NUAK2
TAK-700 (Orteronel)	CYP17A1
Loxistatin Acid (E-64C)	cysteine protease
Zibotentan (ZD4054)	EDNRA
Pyrimethamine	DHFR
RKI-1447	ROCK1, ROCK2
UNC2250	MERTK
SMI-4a	PIM1
SB415286	GSK3A
PRT062607 (P505-15, BIIB057) HCl	SYK
Torcetrapib	CETP
MK-2206 2HCl	AKT1, AKT2, AKT3
ML130 (Nodinitib-1)	NOD1
PF-04217903	MET
GW441756	NTRK1
Varespladib (LY315920)	PLA2G2A
ML161	PARP1
MK-2866 (GTx-024)	AR
Ticagrelor	P2RY12
Letrozole	CYP19A1
GW2580	CSF1R
Zosuquidar (LY335979) 3HCl	ABCB1
KU-60019	ATM
LY2228820	MAPK11, MAPK12, MAPK13, MAPK14
MLN2238	PSMC1
Org 27569	CNR1
Oxymetazoline HCl	ADRA1A

DMXAA (Vadimezan)	NQ01
Anacetrapib (MK-0859)	CETP
AM1241	CNR2
Embelin	XIAP
Toremifene Citrate	ESR
GSK2656157	EIF2AK3
Felodipine	CACNA1C
(+)-Bicuculline	GABR, KCNMA1
Ticlopidine HCl	P2RY
SANT-1	SMO
Ispinesib (SB-715992)	kinesin spindle protein (KSP)
BTZ043 Racemate	decaprenylphosphoryl-β-D-ribose 2'-epimerase (DprE1)
AZD7762	CHEK1, CHEK2
AVL-292	BTK
Pimobendan	PDE3
DBeQ	VCP
Formoterol Hemifumarate	ADRB2
CNX-774	BTK
Lovastatin	HMGCR
4μ8C	ERN1
Lafutidine	HRH2
AZ191	DYRK1B
(-)-Parthenolide	MDM2, P53
JSH-23	NFKB
Pramipexole	DRD2S,DRD2L, DRD3, DRD4
RepSox	TGFBR1
Bazedoxifene HCl	ESR1, ESR2
Golgicide A	GBF1
LDE225 (NVP-LDE225,Erismodegib)	SMO
Ridaforolimus (Deforolimus, MK-8669)	MTOR
LY2784544	JAK2
SNS-314 Mesylate	AURKA, AURKB, AURKC
BGJ398 (NVP-BGJ398)	FGFR1, FGFR2, FGFR3
Irinotecan	TOP1
OSI-420	EGFR
Dutasteride	SRD5A2, SRD5A1
Apigenin	CYP2C9
Rigosertib (ON-01910)	PLK1, PLK2
Forskolin	ADCY4
Rolipram	PDE4
Bupivacaine HCl	ADCY4
UNC669	L3MBTL1
Tioxolone	CA1
PF-4708671	RPS6KB1
5-hydroxymethyl Tolterodine (PNU 200577, 5-HMT, 5-HM)	CHRM1
XAV-939	WNT
SB742457	HTR6
Cinacalcet HCl	CASR
Linagliptin	DPP4
Etomidate	GABR
Entacapone	COMT
AG-14361	PARP1
Moclobemide (Ro 111163)	MAOA
LY411575	APP, NOTCH
GDC-0152	XIAP, BIRC7, AP1, AP2
OC000459	PTGDR2

NLG919	IDO1
Levosulpiride	DRD2
Imatinib (STI571)	ABL1, KIT, PDGFR
DCC-2036 (Rebastinib)	ABL1, SRC, LYN, FGR, HCK, KDR, FLT3
XL335	NR1H4
Nilvadipine	CACNA1C
CHIR-98014	GSK3A, GSK3B
GW4064	NR1H4
PF-5274857	SMO
GDC-0068	AKT1, AKT2, AKT3
JNJ-1661010	FAAH
VU 0364770	GRM4
U-104	CA12
Daunorubicin HCl	TOP2
PF-562271	PTK2
AZD3463	ALK, IGF1R
IOX2	EGLN1
IMD 0354	IKBKB, CHUK
CRT0044876	APE1
TCID	UCHL3
LB42708	FNTA
Necrostatin-1	RIPK1
Empagliflozin (BI 10773)	SLC5A2
SU11274	MET
Bortezomib (PS-341)	PSMC1
YM155 (Sepantronium Bromide)	BIRC5
Lenalidomide (CC-5013)	TNF
Ivacaftor (VX-770)	CFTR
AUY922 (NVP-AUY922)	HSP90A, HSP90B
Agomelatine	HTR2C
17-AAG (Tanespimycin)	HSP90
SP600125	JKAMP, MAPK9, MAPK10; MAP2K4; MAP2K3, MAP2K6, AKT1, PRKC; MAPK1, MAPK14, CHEK1, EGFR
CEP-18770 (Delanzomib)	PSMC1
Aprepitant	TACR1
Fluvoxamine maleate	serotonin (5-HT) reuptake inhibitor (SSRI)
Oligomycin A	ATPAF1
Ginkgolide A	GABR
Cryptotanshinone	STAT3
ICG-001	WNT
Stattic	STAT3
SC144	IL6ST
SRPIN340	SRPK1
Trelagliptin	DPP4
Panobinostat (LBH589)	HDAC
VX-680 (Tozasertib, MK-0457)	AURKA
GDC-0941	PIK3CA, PIK3CD
OSU-03012 (AR-12)	PDK1
GSK690693	Akt1, PKCη, PKCθ, PrkX, Akt3, Akt2, PKCδ, PKCβ, PKCε, PKA, PKG1β
Everolimus (RAD001)	MTOR
MK-8245	SCD
Aniracetam	GRIA1,
Doxazosin Mesylate	ADRA1A
Ginkgolide B	PTAFR
Tosedostat (CHR2797)	LAP3, NPEPPS, ANPEP
Rebamipide	CCKAR
Rasagiline Mesylate	MAOB
PD128907 HCl	DDR3

Apatinib	KDR
ADX-47273	GRM5
AZ 3146	TTK, CENPE
VU 0357121	GRM5
(-)-MK 801 Maleate	GRIN
Mirabegron	ADRB3
AP26113	ALK
Birinapant	DIABLO (AP1)
AZD1981	PTGDR2
LDK378	ALK
(S)-crizotinib	NUDT1
ZM 447439	AURKA, AURKB
BX-912	PDK1
Tadalafil	PDE5
Elvitegravir (GS-9137, JTK-303)	integrase inhibitor
Fostamatinib (R788)	SYK
GSK J4 HCl	KDM6A, KDM6B
TCS 359	FLT3
Carvedilol	ADRB1
Naftopidil	ADRA1A
ML133 HCl	KCNJ2
T0070907	PPARG
Gliquidone	KCNJ
SC-514	IKBKB
ZCL278	CDC42
Caffeic Acid Phenethyl Ester	NFKB
VU 0364439	GRM4
SB431542	TGFBR1
Odanacatib (MK-0822)	CTSK
Celecoxib	PTGS2
Etodolac	PTGS1
Isotretinoin	NA
Stavudine (d4T)	reverse transcriptase inhibitor (NARTI)
VX-745	MAPK14
GSK429286A	ROCK1, ROCK2
SB408124	HCRTR1
H 89 2HCl	PRKAC
Mubritinib (TAK 165)	ERBB2
BMS-378806	CD4
Ki16198	LPAR1, LPAR3
AZ20	ATR
AMG-517	TRPV1
NMS-873	VCP
Sorafenib	RAF1, BRAF, KDR
NH125	EEF2K, PRKC, PRKA, CAMK2, Histidine Kinase
Sal003	EIF2A
Tariquidar	ABCB1
Lomeguatrib	MGMT
BI 2536	PLK1, PLK2, PLK3
Imidapril HCl	ACE
GSK461364	PLK1, PLK2, PLK3
Gliclazide	KCNJ
Sotrastaurin	PRKC (especially PRKCQ; inactive to PRKCZ)
BI-D1870	RPS6KA1, RPS6KA2, RPS6KA3, RPS6KA4
Go 6983	PRKCA, PRKCB, PRKCG, PRKCD
MNS (3,4-Methylenedioxy-β-nitrostyrene, MDBN)	SYK, SRC, VCP
THZ1	CDK7

Table S8: 40 drugs used for flow cytometry based HTS	
**Compounds**	**Targets**
Selumetinib (AZD6244)	MAP2K1, MAPK3, MAPK1
Stattic	STAT3
CYCLOPHOSPHAMMIDE	alkylating agent
CHIDAMIDE	HDAC1, 2, 3, and 10
Trichostatin A (TSA)	ALL HDACS (Except HDAC8)
Semagacestat (LY450139)	APP, NOTCH
TGR1202	PI3Kδ
CRIZOTINIB	c-Met and ALK
IDELASIB	p110δ
17-AAG (Tanespimycin)	HSP90
DECITABINE	DNA methyltransferase
NVP-ADW742	IGF1R
Enzastaurin (LY317615)	PRKCB, PRKCA, PRKCG, PRKCE
RO4929097	γ secretase (NOTCH)
DEXAMETASONE	glucocorticoid
Crenolanib (CP-868596)	PDGFRA, PDGFRB, FLT3 (D842V)
Belinostat (PXD101)	HDAC
Tofacitinib (CP-690550,Tasocitinib)	JAK3
PRALATREXATE	antifolate
GDC-0068	AKT1, AKT2, AKT3
Ruxolitinib (INCB018424)	JAK1, JAK2
SC144	IL6ST
DAUNORUBICIN	inhibits DNA synthesis
VINCRISTINE	inhibitor of polymerization of microtubules
MK-1775	WEE1
KPT-330	XPO1
CHIR-124	CHEK1
Ganetespib (STA-9090)	HSP90
ABT-263 (Navitoclax)	BCL2, BCL2L1, BCL2L2
AUY922 (NVP-AUY922)	HSP90A, HSP90B
Panobinostat (LBH589)	HDAC
NSC 319726	TP53 (R175)
SN-38	TOP1
THZ1	CDK7
Bortezomib (PS-341)	PSMC1
CEP-18770 (Delanzomib)	PSMC1
MLN2238	PSMC1
Ouabain	ATP1B
ROMIDEPSIN	HDAC1 and HDAC2
YM155 (Sepantronium Bromide)	BIRC5
